# Third-party cytomegalovirus-specific T cells improved survival in refractory cytomegalovirus viremia after hematopoietic transplant

**DOI:** 10.1172/JCI165476

**Published:** 2023-05-15

**Authors:** Susan E. Prockop, Aisha Hasan, Ekaterina Doubrovina, Parastoo B. Dahi, Irene Rodriguez-Sanchez, Michael Curry, Audrey Mauguen, Genovefa A. Papanicolaou, Yiqi Su, JinJuan Yao, Maria Arcila, Farid Boulad, Hugo Castro-Malaspina, Christina Cho, Kevin J. Curran, Sergio Giralt, Nancy A. Kernan, Guenther Koehne, Ann Jakubowski, Esperanza Papadopoulos, Miguel-Angel Perales, Ioannis Politikos, Keith Price, Annamalai Selvakumar, Craig S. Sauter, Roni Tamari, Teresa Vizconde, James W. Young, Richard J. O’Reilly

**Affiliations:** 1 Stem Cell Transplantation and Cellular Therapies Service, Department of Pediatrics, and; 2 Department of Pediatrics, Memorial Sloan Kettering Cancer Center (MSKCC), New York, New York, USA.; 3 Stem Cell Transplant Service, Division of Hematology/Oncology, Department of Pediatrics, Boston Children’s Hospital, Boston, Massachusetts, USA.; 4 Department of Pediatrics Dana-Farber Cancer Institute, Boston, Massachusetts, USA.; 5 Adult Bone Marrow Transplant Service, Department of Medicine, MSKCC, New York, New York, USA.; 6 Department of Medicine, Weill Cornell Medical College, New York, New York, USA.; 7 Department of Epidemiology and Biostatistics,; 8 Infectious Disease Service, Department of Medicine, and; 9 Department of Pathology, MSKCC, New York, New York, USA.; 10 Department of Pediatrics, Weill Cornell Medical College, New York, New York, USA.; 11 Miami Cancer Institute, Baptist Health South Florida, Miami, Florida, USA.; 12 Blood and Marrow Transplant Program, Taussig Cancer Institute, Cleveland Clinic, Cleveland, Ohio, USA.

**Keywords:** Infectious disease, Transplantation, Immunotherapy, Stem cell transplantation, T cells

## Abstract

**Background:**

Refractory CMV viremia and disease are associated with significant morbidity and mortality in recipients of hematopoietic stem cell transplant (HCT).

**Methods:**

In phase I/II trials, we treated 67 subjects for CMV viremia or disease arising after HCT with adoptive transfer of banked, third-party, CMVpp65-sensitized T cells (CMVpp65-VSTs). All were evaluable for toxicity and 59 for response. Evaluable subjects had CMV disease or persisting viremia that had failed at least 2 weeks of induction therapy with a median of 3 antiviral drugs; 84.7% had more than 3 of 11 high-risk features. CMVpp65-VSTs were specific for 1 to 3 CMVpp65 epitopes, presented by a limited set of HLA class I or II alleles, and were selected based on high-resolution HLA matching at 2 of 10 HLA alleles and matching for subject and subject’s HCT donor for 1 or more alleles through which the CMVpp65-VSTs were restricted.

**Results:**

T cell infusions were well tolerated. Of 59 subjects evaluable for response, 38 (64%) achieved complete or durable partial responses.

**Conclusions:**

Recipients responding to CMVpp65VSTs experienced an improved overall survival. Of the risk factors evaluated, transplant type, recipient CD4^+^ and CD8^+^ T cell levels prior to adoptive therapy, and the HLA restriction of CMVpp65-VSTs infused each significantly affected responses. In addition, CMVpp65-specific T cells of HCT donor or recipient origin contributed to the durability of both complete and partial responses.

**Trial Registration:**

NCT00674648; NCT01646645; NCT02136797 (NIH).

**Funding:**

NIH (P01 CA23766, R21 CA162002 and P30 CA008748); Aubrey Fund; Claire Tow Foundation; Major Family Foundation; “Rick” Eisemann Pediatric Research Fund; Banbury Foundation; Edith Robertson Foundation; Larry Smead Foundation.

## Introduction

Cytomegalovirus (CMV) infections remain a major cause of morbidity and mortality following allogeneic hematopoietic stem cell transplants (HCTs) (1–5). Although prophylactic or preemptive treatment with ganciclovir and/or foscarnet and, more recently, prophylaxis with letermovir have reduced the incidence and mortality of early CMV infections (6–8), even with letermovir prophylaxis, 37.5% of subjects reactivate CMV and are at risk for organ toxicity from antiviral agents as well as refractory CMV. Prolonged CMV and CMV-directed therapy may also delay recovery of virus-specific immune responses, predisposing to late-onset disease (6, 7).

Riddell et al. (9, 10) first demonstrated that adoptive transfer of donor-derived CD8^+^ CMV-specific cytotoxic T lymphocyte (CMV-CTL) clones sensitized with autologous CMV-infected fibroblasts could protect HCT recipients from CMV reactivation. Subsequent studies employing HCT donor–derived CMV-specific T cell lines (11–16) also documented the potential of CMV-specific T cells to treat CMV disease. However, generation of HCT donor–derived CMV-specific T cells is time consuming, logistically challenging, and thus often not feasible for subjects in need. We and others are exploring CMV-specific T cells generated from healthy individuals other than the subject’s HCT donor. The therapeutic potential of this approach was first demonstrated in the treatment of EBV posttransplant lymphoproliferative disease (EBV-PTLD) with banked third-party EBV-specific CTLs (EBV-CTLs) (16, 17). However, experience in the treatment of CMV is more limited and factors influencing responses less well defined (5, 18–24).

Here we report the safety and antiviral activity of banked third-party donor–derived CMVpp65-sensitized T cells (CMVpp65-VSTs) of defined epitope specificity and HLA restriction in 67 HCT recipients treated in the preletermovir era. As in other trials evaluating the efficacy of novel antivirals, eligibility for treatment required invasive CMV disease or CMV viremia persisting despite at least 2 weeks of antiviral drugs. However, in this series, most of the subjects were hospitalized with extensively treated, refractory CMV infections. This permitted unique assessment of attributes of recipients treated and the CMVpp65-VSTs administered that were associated with effective and sustained clearance or continued progression of disease.

## Results

### Subject status prior to infusion of CMVpp65-VSTs.

**Figure 1:**
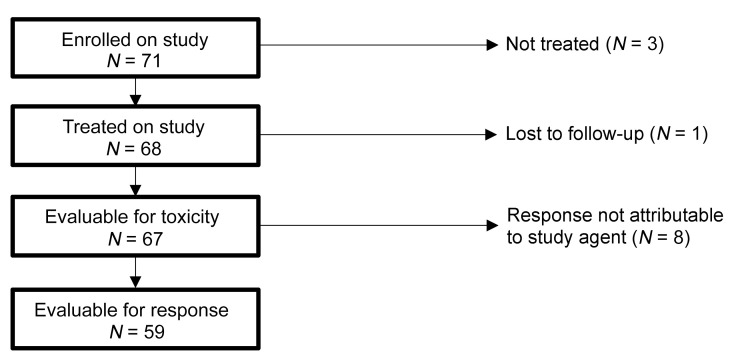
Diagram of patient enrollment, treatment, and evaluability. Patients are reported from 3 IRB-approved studies (ClinicalTrials.gov NCT00674648, NCT01646645, and NCT02136797). Recipients who achieved a CR or PR but had changes made to antiviral therapy just prior to initiation of CMVpp65-VST treatment (*n* = 3) or during treatment on study (*n* = 5) for reasons other than progression of disease were consider nonevaluable for response.

Of 71 subjects enrolled, 3 were not treated; 1 received 1 dose of CMVpp65-VSTs and was lost to follow-up. The remaining 67 subjects were evaluated for toxicity and 59 for efficacy (Figure 1). In the other 8 subjects, responses to CMVpp65-VSTs were considered nonevaluable due to changes in antiviral drug therapy immediately prior to (*n* = 3) or during therapy (*n* = 5). The 67 recipients of third-party donor–derived CMVpp65-VSTs and their responses are described in Supplemental Tables 1–3 (supplemental material available online with this article; https://doi.org/10.1172/JCI165476DS1) and summarized in Table 1. The characteristics of the 59 subjects evaluable for response and the 8 not evaluable were similar (Table 1 and Supplemental Tables 1–3).

The subjects in this trial exhibited features associated with a high risk of CMV-associated mortality (25–29). These are summarized in Table 1. Of the 59 subjects evaluable for response, 38 (64%) received HLA nonidentical transplants, 39 (66%) received T cell–depleted (TCD) grafts, and 6 (10%) received cord-blood transplants.

Additionally, 57 were treated a median of 140 (range 29–584) days after HCT and a median of 97 days (range 7–564) after reactivating CMV. One subject was treated over 10 years (4,940 days) and 1 over 5 years (1,954 days) following HCT after donor lymphocyte infusion (DLI) and prolonged therapy for chronic graft-versus-host disease (GVHD), respectively. Only 9 were treated fewer than 30 days after reactivation of CMV.

Of the 59 evaluable subjects, 20 were treated for biopsy-proven invasive CMV disease. These included 10 with CMV enteritis, 6 with CMV meningoencephalitis and/or chorioretinitis, 2 with both enteritis and CNS disease, and 2 subjects, including 1 in respiratory failure, with CMV pneumonia confirmed by bronchoalveolar lavage (BAL). The other 39 subjects were treated for persistent CMV viremia without biopsy-proven invasive disease. Of these 39, 6 as well as 3 with CMV disease (enteritis) had bilateral interstitial infiltrates by CAT scan, but without a BAL to confirm CMV pneumonia. Of the 8 subjects not evaluable for response, 1 was treated for CMV pneumonia and 7 had viremia, of whom 2 also had bilateral lung infiltrates.

The 59 evaluable subjects had received a median of 3 lines of prior therapy with antiviral drugs including ganciclovir and/or valganciclovir (*n* = 55), foscarnet (*n* = 51), and cidofovir or brincidofovir (*n* = 19). Nineteen subjects had received all 3 antiviral agents; only 7 had received a single antiviral agent prior to CMVpp65-VST therapy. Additional CMV-directed therapy included marabavir (*n* = 3), leflunamide (*n* = 4), CMV immune globulin (*n* = 17), and primary HCT donor–derived CMVpp65-VSTs (*n* = 1) (Supplemental Table 1). The cumulative CMV viral load by time-averaged AUC (AAUC) was calculated for 24 subjects and was in the previously validated high-risk quartile (CMV AAUC >1.5) in 75% of cases (Supplemental Table 3) (26). Furthermore, in 28 of 49 subjects tested (56%), the CMV isolated had mutations conferring drug resistance to ganciclovir (*n* = 28), foscarnet (*n* = 15), and cidofovir (*n* = 8).

Based on 6 risk factors reported for CMV mortality (25–30), HLA disparity, TCD HCT, number of antivirals, duration of prior treatment, cumulative viral load, and drug resistance mutations, 50 of 59 subjects (84.7%) had more than 3 risk factors prior to treatment with CMVpp65-VSTs (Supplemental Table 3).

### Characterization of T cells infused.

**Figure 2:**
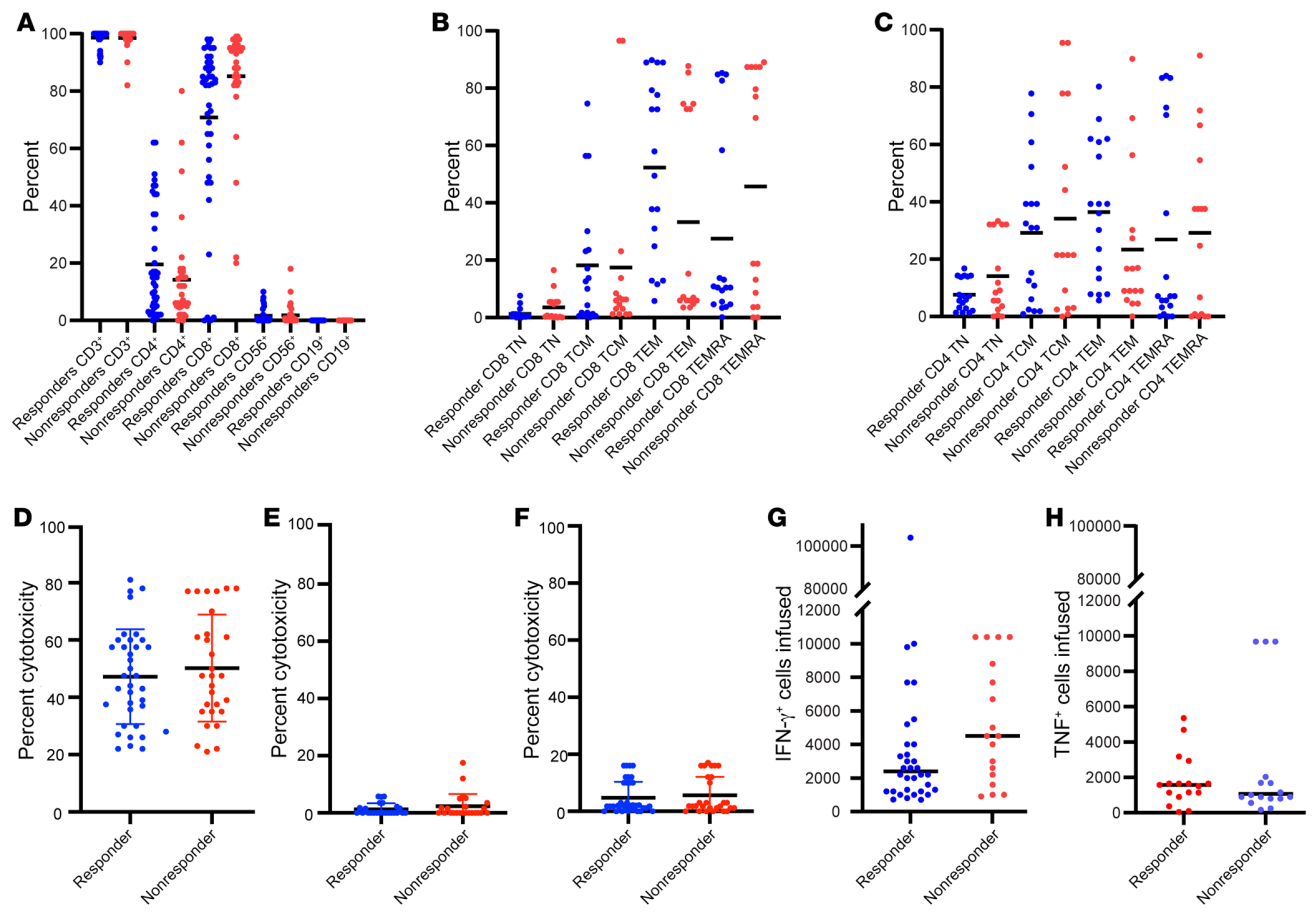
Phenotype and cytotoxicity of CMVpp65VSTs administered. Phenotype of CMVpp65VSTs administered to subjects achieving a CR or PR (blue) compared with those administered to subjects not achieving a CR or PR (red) as analyzed by Wilcoxon’s rank-sum test. Lines used on multiple occasions are represented multiple times. (**A**) From left: percentages of CD3^+^ (*P* = 0.96), CD3^+^CD4^+^ (*P* = 0.24), CD3^+^CD8^+^ (*P* = 0.35), CD56^+^16^+^ NK (*P* = 0.99), and CD19^+^ B cells (*P* = 0.77). *n* = 77. (**B**) From left: percentages of CD8^+^ TN (*P* = 0.88), CD8^+^ TCM (*P* = 0.78), CD8^+^ TEM (*P* = 0.35), and CD8^+^ TEMRA (*P* = 0.28). *n* = 34. (**C**) From left: percentages of CD4^+^ TN (*P* = 0.92), CD4^+^ TCM (*P* = 0.88), CD4^+^ TEM (*P* = 0.99), and CD4^+^ TEMRA (*P* = 0.96). *n* = 34. Cr release assay demonstrating cytotoxicity of infused CMVpp65 lines against (**D**) autologous PHA blasts loaded with (*P* = 0.49) or (**E**) autologous PHA blasts not loaded with CMVpp65 peptides (*P* = 0.29). *n* = 65 (**D**); *n* = 65 (**E**). (**F**) Allogeneic PHA blasts not loaded with CMVpp65 peptides (*P* = 0.60). *n* = 65. (**G**) Absolute number of IFN-γ–producing cells per 10^5^ CMVpp65VSTs infused (*P* = 0.59). *n* = 52. (**H**) Absolute number of TNF-α–producing cells per 10^5^ CMVpp65VSTs infused (*P* = 0.49). *n* = 35. No significant differences in the CMVpp65VSTs administered to responding versus nonresponding patients were observed.

We administered T cells from 48 of 138 CMVpp65CTL lines in our bank. The CMVpp65-VSTs infused were predominantly CD3^+^CD8^+^; only 6 lines had more than 50% CD3^+^CD4^+^ T cells (Figure 2A). We had samples sufficient to further analyze CMVpp65-VSTs administered to 39 of the 59 evaluable subjects. As shown in Figure 2, B and C, the T cells were predominantly CD8^+^ effector memory T cells (TEM) and CD8^+^ effector memory cells reexpressing CD45RA (TEMRA). The CD8^+^ and CD4^+^ T cell populations contained variable proportions of central memory T cells (TCM) and naive (TN) T cells. All lines lysed CMVpp65 peptide pool–loaded autologous phytohaemagglutinin (PHA) blasts (Figure 2, D and C), but not autologous or allogeneic PHA blasts alone (Figure 2, E and F). The concentration of CD3^+^IFN-γ^+^ T cells responding to CMVpp65 peptides varied from 720 to 88,000/10^6^ T cells, with a median of 22,000/10^6^ T cells administered (Figure 2G). There were no significant differences in the phenotype or function of T cells administered to subjects who responded to therapy compared with those who did not respond to therapy. As shown in Figure 2H, the CMVpp65-VSTs administered also contained proportions of TNF-α–secreting T cells. Again, there were no significant differences detected between the CMVpp65-VSTs administered to those who responded compared with those who did not.

Each CMVpp65-VST line administered was restricted by an HLA allele shared by both subject and HCT donor and was matched to the subject for 2 or more HLA alleles. As detailed in Table 2, the specificities of CMVpp65-VSTs infused included 19 CMVpp65 peptide epitopes presented by 25 HLA alleles. CMVpp65-VSTs specific for epitopes presented by HLA A0201, B0702, B3501, and B0801 were the most frequently used.

### Toxicities and adverse events.

Infusions were well tolerated. No subject experienced fever or other toxicities over the first 48 hours of observation. Nine subjects had 21 possibly related adverse events; 19 of these were grade 3 or higher (Table 3) and none were probably or definitely related to infusion of CMVpp65-VSTs. The only recurrent possibly related adverse events were respiratory in 6 subjects developing 7 to 34 days after the first infusion of CMVpp65-VSTs. Of these 6 subjects, 2 had previously radiographically documented CMV pneumonia with BAL documentation of CMV 27 and 29 days after first infusion (unique patient number [UPN] 22532 and UPN 5560), and 1 had viremia and CNS disease at baseline with BAL confirmation of CMV 22 days after first infusion (UPN 4193). Of the other 3, 1 had recurrent diffuse alveolar hemorrhage (UPN 5066), 1 had hypoxia with sepsis (UPN 4062), and 1 had transient tachypnea due to fluid overload (UPN 21968).

Importantly, 3 other patients had BAL-documented CMV pneumonia and 7 had infiltrates concerning for CMV pneumonia and did not experience respiratory events. One of these 7 patients (UPN 22174) with abnormal imaging at baseline experienced progression of long-standing idiopathic pneumonia syndrome with a biopsy negative for CMV considered unrelated to CMVpp65-VSTs. Of the 67 patients, 26 had a history of GVHD, including 13 on low-dose (<0.5 mg/kg) corticosteroids at the start of therapy. None of these 26 patients had a flare or recurrence of GVHD. However, one subject developed cytopenia and de novo grade 3 GVHD (UPN 5335) 42 days after the last dose of CMVpp65-VSTs and in conjunction with reactivation of HHV6 infection.

### Clinical outcomes.

Of 59 subjects evaluable, 38 (64.4%) responded to CMVpp65-VSTs; 20 achieved complete response (CR) and 18 partial response (PR). Clearance or a 2log_10_ reduction in levels of CMV was observed in 26 of 39 evaluable subjects (67%) treated for viremia alone (14 CR; 12 PR) and in 12 of 20 (60%) treated for CMV disease (6 CR; 6 PR) (Table 4). Of 2 with documented CMV pneumonia, 1 in respiratory failure at baseline died and 1 recovered (CR). Of 9 viremic subjects with baseline interstitial infiltrates consistent with CMV pneumonia, 6 recovered (2 CR; 4 PR). Of 8 subjects with documented CMV meningoencephalitis with or without chorioretinitis, 3 achieved a CR and 3 a PR; all 6 ultimately cleared and are long-term survivors. In addition, of 28 with drug-resistant CMV evaluable for response, 18 (64%) achieved a CR or PR following treatment with CMVpp65-VSTs (Table 5).

Importantly, the patients with CRs (*n* = 20) and all but 1 of the 18 with PRs persisted for at least 6 months after completion of therapy (*n* = 12), or until removal from study due to DLI for leukemic relapse (*n* = 1) or death due to other causes (*n* = 4). The 12 subjects with PRs lasting 6 or more months included 6 subjects who achieved PR based on resolution of symptoms of organ-based disease and clearance of viremia without biopsy to document CR and 6 subjects who had a 2log_10_ or greater reduction in CMV DNA levels and continued with intermittent low-grade viremia not requiring reinstitution of antiviral therapy. 

**Figure 3:**
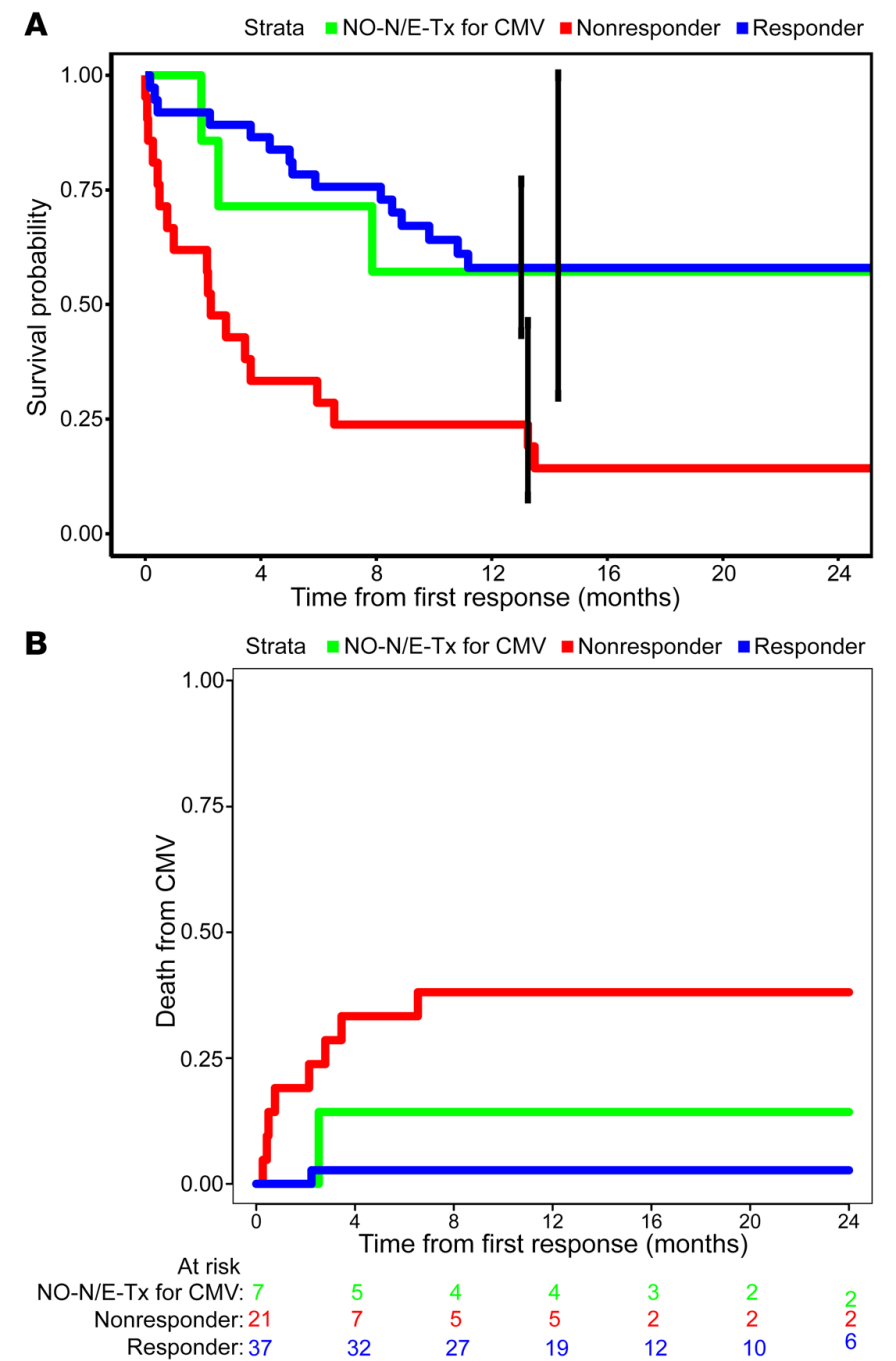
OS and cumulative incidence of CMV-related deaths in subjects responding and not responding to CMVpp65VST therapy. Kaplan-Meier OS (**A**) and Aalen-Johnson estimates of the cumulative incidence of CMV-related death (**B**) in recipients of CMVpp65-VSTs responding to (blue) and not responding to therapy (red) as well as those not evaluable (NO-N/E-Tx) (green) for response. Responders (blue) had longer OS compared with nonresponders (red) (log-rank test *P* < 0.001), but there was no difference between responders and nonevaluable (green) subjects (log-rank test, *P* = 0.9). Nonresponders died more of CMV than responders (Gray’s test, *P* = 0.001), and there was no difference between nonevaluable subjects and responders (Gray’s test, *P* = 0.42).

The CRs and PRs resulted in a significant reduction in CMV-related mortality as well as an overall survival (OS) advantage (Figure 3). Of 38 responders, only 1 (UPN 5073), treated for viremia alone, died of CMV after developing CMV pneumonia 60 days after completing therapy. OS for responders was 79% and 58% at 6 months and 2 years, respectively (Figure 3A). In contrast, 40% of the nonresponders succumbed to CMV (*n* = 6) or toxic sequelae of subsequent drug treatment (*n* = 2); their OS at 6 months was 29% and at 2 years was 14% (*P* < 0.001) (Figure 3B).

### Baseline characteristics associated with clinical response.

We analyzed whether specific subject characteristics (Table 5) or characteristics of CMV infection (Table 6) prior to CMVpp65-VSTs were predictive of response, focusing on features associated with increased CMV-induced mortality (26–30). As shown in Table 6, recipients of unmodified HCTs had a higher response rate (93%) than recipients of TCD grafts (56%) (*P* = 0.02) or TCD and cord-blood grafts combined (55%) (*P* = 0.01). The response rate for recipients of HLA-matched HCTs was not significantly higher than for subjects who received HLA-nonidentical grafts (73% vs 59%, *P* = 0.42). There were no differences between responders and nonresponders in ongoing immunosuppression for GVHD prophylaxis. However, we did find that the numbers of both CD4^+^ (*P* = 0.004) (31) and CD8^+^CD3^+^ T cells (*P* = 0.005) in the blood at baseline were significant predictors of subsequent response (30).

Transfer of CMV-reactive T cells from a seropositive HCT donor in an unmodified marrow graft might be expected to add to the antiviral effects of the third-party CMVpp65-VSTs infused. However, in this cohort who predominantly received TCD (*n* = 39) or cord blood (*n* = 6) grafts, we found no difference in the rate of response between recipients of transplants from CMV-seropositive versus seronegative donors. Conversely, the unexpected albeit insignificantly higher response rate observed in recipients of transplants from CMV-seronegative donors in this cohort likely reflects the distribution of CMV-seronegative donors (9 of 11 documented) for the 14 recipients of unmodified transplants in this cohort. Only 1 of these patients failed to respond to the CMVpp65-VSTs.

As shown in Table 5, analysis of the CMV infections and their treatment prior to infusions of CMVpp65-VSTs revealed no significant differences between responders and nonresponders in terms of the severity of CMV infection (i.e., organ diseases versus persistent viremia), time from transplant or CMV reactivation to treatment with CMVpp65-VSTs (28), the number of prior antiviral agents received, infection with a CMV strain genetically resistant to CMV-directed antiviral drugs (29), or the cumulative CMV viral load as estimated by the AAUC (26, 30). However, although there was no difference between responders and nonresponders in the number of antiviral agents received prior to initiation of CMVpp65-VST infusions, there was a lower response rate among individuals receiving ganciclovir immediately prior to and during treatment (20/37; 54%) compared with subjects receiving other antiviral drugs (18/22; 82%), (*P* = 0.04). Although ganciclovir has been reported to delay recovery of T cell responses after transplant, there was no difference in the proportion of recipients with a baseline CD4 count greater than 50 in those receiving versus not receiving ganciclovir (11/35 versus 13/32; *P* = 0.81) or in the time from CMV reactivation to first infusion (median of 163 versus 167 days).

CMVpp65-VSTs administered to responders did not differ significantly from those given to nonresponders in their content of CD4^+^ or CD8^+^ T cells, IFN-γ^+^ CMVpp65 peptide pool–responsive CD3^+^ T cells, or TNF-α^+^ CD3^+^ T cells (Figure 2). They also exhibited similar cytotoxicity against CMVpp65 peptide–loaded autologous cytokine-activated monocytes (CAMS) (Figure 2). Notably, response did not increase as the number of HLA alleles matched between subject and third-party donor increased (*P* = 0.23). Furthermore, while CMVpp65-VSTs restricted by more than 1 HLA allele shared by the subject and HCT donor induced responses in 8 of 11 (72.7%) cases, this response rate did not differ significantly when compared with 30 of 48 (62.5%) recipients of CMVpp65-VSTs restricted by a single shared allele (*P* = 0.14).

In contrast, specific HLA restrictions of the CMVpp65-VSTs did affect outcomes. As shown in Table 7, 18 of 27 (66%) subjects treated with HLA A0201-restricted CMVpp65-VSTs specific for NLVPMVATV responded, as did 3 of 5 treated with CMVpp65-VSTs restricted by the consistently immunodominant HLA-B*0702 that were specific for TPRVTGGGAM or RPHERNGFTY. Similarly, 4 of 5 treated with CMVpp65-VSTs specific for YSEHPTFTSOY presented by HLA*B0801 and HLA*A0101 and 4 of 6 treated with CMVpp65-VSTs specific for the HERNGFTVL epitope presented by HLA B*4001, B*4006, B*4201, and B*4403 responded. In contrast, 0 of 7 subjects treated with CMVpp65-VSTs specific for epitopes presented by HLA B35 allelic variants shared by the subject and HCT donor responded (*P* = 0.001). Of the 8 remaining subjects treated with CMVpp65-VSTs restricted by other class I HLA alleles, all 8 (100%) responded. Only 2 subjects received predominantly CD4^+^ CMVpp65-VSTs restricted by class II HLA alleles; 1 died early with progression, and the other achieved a CR. Thus, the complete lack of responses to CMVpp65-VSTs restricted by HLA B*35 alleles differed significantly from the high response rates achieved with CMVpp65-VSTs restricted by other HLA alleles (*P* = 0.001).

In the light of this finding, we examined other clinical variables that might explain the lack of response to HLA B35–restricted CMVpp65-VSTs. Specifically, we compared these 7 subjects to the other subjects in the evaluable cohort for the presence of risk factors associated with severe CMV disease and CMV mortality. As shown in Supplemental Table 3, the average number of risk factors for recipients of HLA B35–restricted CMVpp65-VSTs (4.57 versus 5.4, *P* = 0.88) and the proportions of them with each risk factor were similar to those of the other 52 subjects in the evaluable cohort, except that they all had received a TCD (*n* = 6) or cord-blood graft (*n* = 1), a high-risk factor, and all had only been treated for persistent viremia. We also compared baseline CD4^+^ T cell counts in recipients of B35-restricted CMVpp65-VSTs (median 35.9 CD4^+^ T cells/μL) to those in recipients of CMVpp65-VSTs restricted by other HLA alleles (median 64.4 CD4^+^ T cell/μL), but this difference was also not significant (*P* = 0.4).

### Alterations in circulating levels of CMV DNA and CMVpp65-VSTs induced following adoptive T cell transfer.

**Figure 4:**
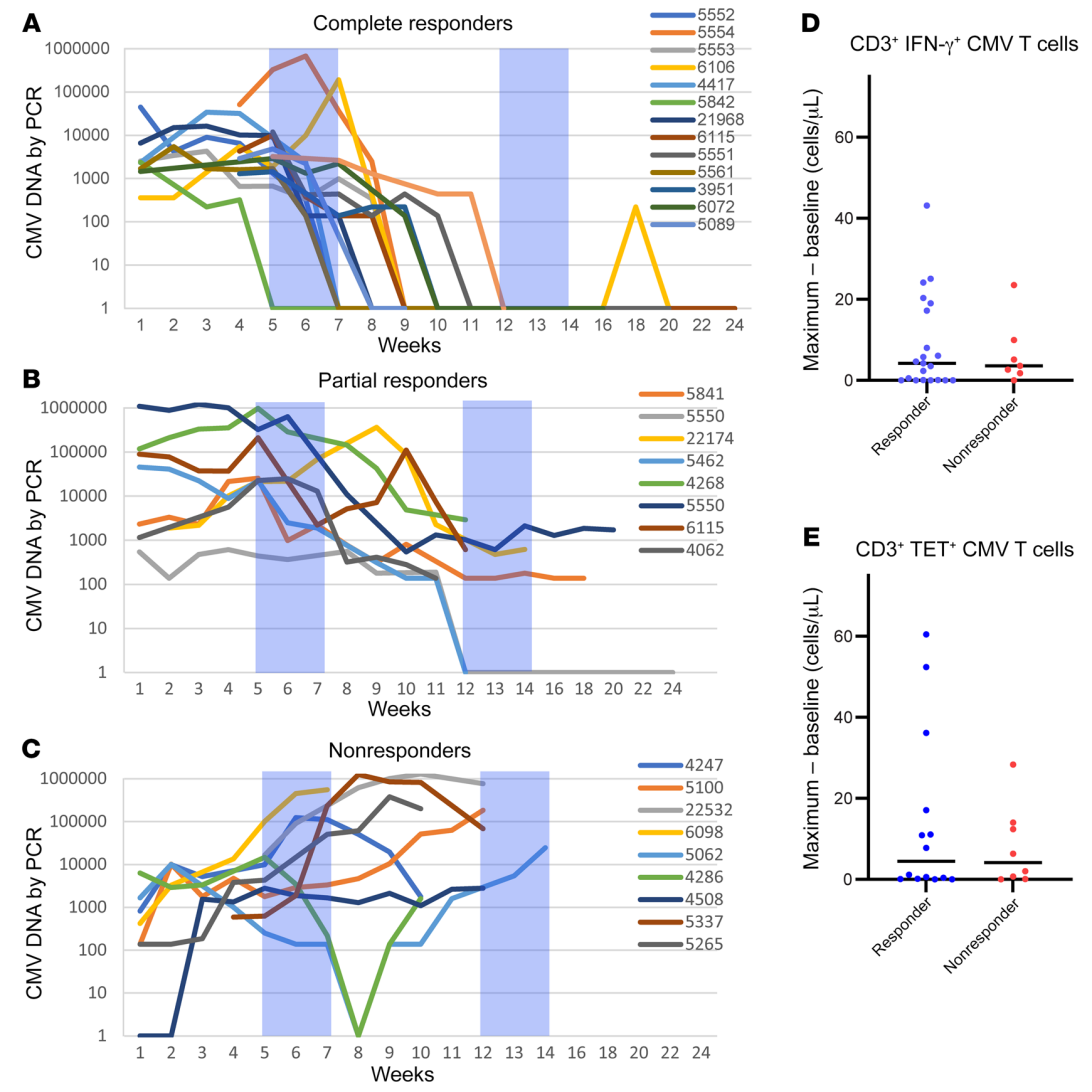
Alterations of CMV viremia and CMV-specific IFN-γ^+^CD3^+^ T cells and tetramer^+^ CMVpp65VSTs in treated subjects. Weekly CMV PCR measurements in subjects achieving (**A**) CR, (**B**) PR, and (**C**) not responding to therapy. Shaded bars represent time of response assessment. Maximum expansion of CMV-specific T cells isolated from the blood of treated subjects responding to or not responding to therapy was measured by IFN-γ and tetramer by subtracting the baseline number from the peak. (**D**) Number of CMVpp65-specific T cells (identified as IFN-γ^+^CD3^+^) in recipients of third-party CMVpp65VSTs responding to (blue) and not responding to (red) therapy. CD3^+^IFN-γ^+^ T cell numbers were calculated as a fraction of CD3^+^ T cells/μL. The absolute increase in the number of CD3^+^IFN-γ^+^ T cells (i.e., maximum minus baseline) identified in each subject within 70 days of infusion is plotted. The maximum increase of CMVpp65-specific CD3^+^IFN-γ^+^ T cells was not different in responders compared with nonresponders (*P* = 0.96). *n* = 28. (**E**) Number of CMVpp65-specific T cells (identified as TET^+^CD3^+^) in recipients of third-party CMVpp65VSTs responding to (blue) and not responding to (red) therapy. CD3^+^TET^+^ T cell numbers were calculated as a fraction of CD3^+^ T cells/μL. The absolute increase in the number of CD3^+^TET^+^ T cells identified in each subject within 70 days of infusion is plotted. The maximum increase in the number of CMVpp65-specific CD3^+^TET^+^ T cells was also not different in responders compared with nonresponders (*P* = 0.99). *n* = 21. Comparisons of the increases in CD3^+^IFN-γ^+^ T cells and TET^+^ T cells were analyzed using Wilcoxon’s rank-sum test.

Sequential measurements of CMV DNA in the blood were used to monitor responses and provided a means for distinguishing not only responding subjects from treatment failures, but also those with CRs versus PRs. As illustrated in Figure 4A, CMV DNA levels increased in most subjects in the week after the first CMVpp65-VST infusion, irrespective of subsequent response. However, in subjects who achieved a CR, CMV DNA levels subsequently fell dramatically, with complete clearance by the end of the first or second cycle of CMVpp65CTL infusions. Subjects with a PR (Figure 4B) experienced 2log_10_ or greater eductions of CMV DNA from peak levels over the same time period. They continued to have intermittent low levels of CMV DNA, but did not require reinstitution of antiviral drugs. In contrast, in those who failed to respond (Figure 4C), CMV DNA levels either continued to rise or in 2 cases, transiently fell but had increased again by the next weekly testing.

At the same intervals, we tracked blood levels of CMVpp65-specific CD3^+^IFN-γ^+^ T cells in 29 subjects and T cells binding the specific CMVpp65 peptide HLA tetramers targeted by the infused CMVpp65-VSTs in 23 subjects after CMVpp65-VST infusions. Increments in CMVpp65-specific IFN-γ^+^ T cells and TET^+^ T cells were detected in 86% of both responders and nonresponders. However, in the 70 days after initiation of CMVpp65-VST infusions, maximal increases in the levels of CMVpp65-specific IFN-γ^+^ T cells as well as TET^+^ T cells were not significantly higher in responders compared with nonresponders (*P* = 0.96 and *P* = 0.99 respectively; Figure 4, D and E). The basis for this lack of a difference is unclear. It may, in part, reflect the range and non-Gaussian distribution of the increases in CMVpp65-specific IFN-γ^+^ and TET^+^ T cells observed in both the responding and nonresponding groups. Because 13 of 14 recipients of unmodified grafts responded to CMVpp65 VSTs and had higher baseline and peak CMVpp65 IFN-γ^+^ T cells detected in vivo than recipients of TCD HCT, we compared peak expansions of CMVpp65-specific IFN-γ^+^CD3^+^ T cells in the 39 evaluable recipients of TCD grafts. Again, however, we found no significant differences between responders and nonresponders (data not shown).

**Figure 5:**
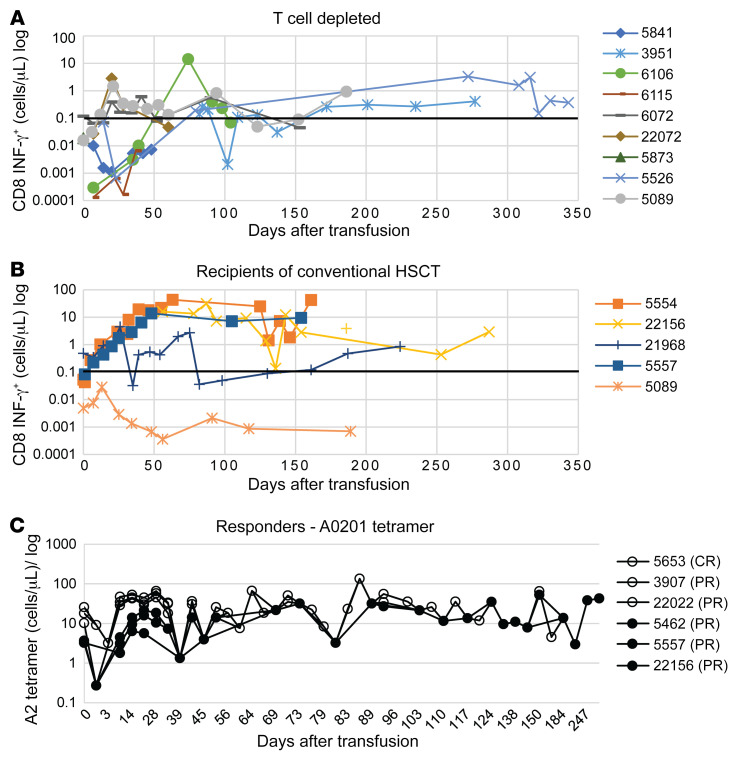
Detection of CMV-specific T cells isolated from responding recipients of conventional versus TCD hematopoietic transplant after adoptive transfer of CMVpp65-VSTs. CMVpp65-specific T cells (identified as IFN-γ^+^CD3^+^) in recipients of third-party CMVpp65VSTs after (**A**) TCD and (**B**) conventional HCT. The number of CD3^+^IFN-γ^+^ T cells were calculated based on their fraction of CD3^+^ T cells/μL. Absolute numbers of CD3^+^IFN-γ^+^ T cells are plotted prior to infusion and serially measured over time. They were compared using Wilcoxon’s rank-sum test. The baseline number of CMVpp65-specific CD3^+^IFN-γ^+^ T cells did not differ between the 2 groups (*P* = 0.025), but the difference in persistence of expansion can also be appreciated by the comparison of measurements above the line at 0.1log_10_. (**C**) Comparison of the in vivo expansion of CD3^+^ T cells binding NLV peptide/HLA A0201 tetramers following adoptive transfer of NLV-specific, HLA A0201-restricted third-party CMVpp65VSTs in responding recipients of unmodified (*n* = 3) or TCD (*n* = 3) HCTs from CMV-seronegative donors. Recipients of unmodified HCTs are depicted by solid circles and lines, those receiving TCD HCTs by open circles.

We specifically evaluated alterations in the number of postinfusion circulating CMVpp65-specific T cells in recipients of transplants from CMV-seronegative donors, since one report had questioned whether third-party T cells actually expand in HCT recipients (23). As shown in Figure 5, A and B, increments in levels of CMVpp65 peptide pool–specific IFN-γ^+^ T cells were detected following the first or second cycle of infusions in recipients of both unmodified and TCD HCTs from seronegative donors. However, baseline levels were lower (*P* = 0.025) and postinfusion levels achieved in the blood of recipients of TCD grafts were lower than those achieved in recipients of unmodified grafts, even though the latter subjects were receiving GVHD prophylaxis with cyclosporine, tacrolimus, or sirolimus. We were also able to measure T cells binding tetramers of the epitope/HLA targeted by the CMVpp65-VSTs in a subset of recipients of transplants from seronegative donors treated with HLA A0201-restricted, NLV-specific CMVpp65-VSTs. In these subjects, levels of NLV/HLA A0201 tetramer^+^ T cells achieved after infusion were very similar (Figure 5C). Taken together, these findings suggest that both the infused CMVCTLs and transplant donor–derived or residual host–derived IFN-γ^+^ T cells specific for other CMVpp65 peptides in the pool are induced to proliferate following CMVpp65CTL infusions.

**Figure 6:**
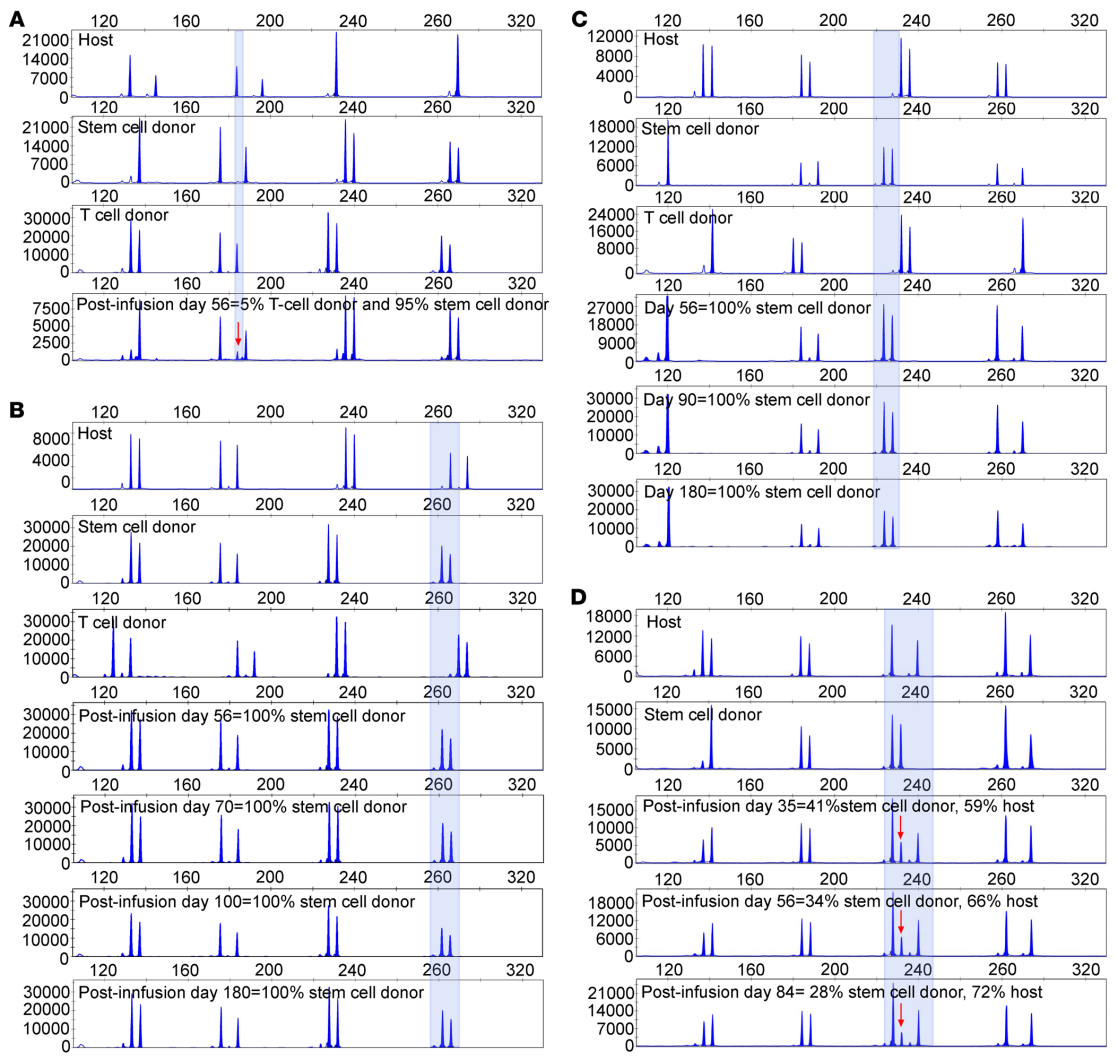
Identification of the origin of CMVpp65-specific T cells circulating after infusion. Four recipients of CMVpp65-VSTs all had undetectable CMV-specific T cells by IFN-γ and/or tetramer at baseline. (**A**) UPN 4417 CMV-seropositive recipient and HCT donor. Unique STR profile for recipient, HCT donor, and third-party donor–derived CMVpp65-VSTs with third-party CMVpp65VSTs identified 56 days after infusion. (**B**) UPN 2386 CMV-seropositive recipient and seronegative HCT donor with identification of HCT donor–derived CMV–specific T cells identified 56 days after initial infusion. (**C**) UPN 3907.CMV-seropositive recipient and seronegative HCT donor treated for isolated CNS disease and with identification of HCT donor–derived CMV-specific T cells identified 56 days after initial infusion. (**D**) UPN 5653 CMV–seropositive recipient and seronegative HCT donor with identification of both recipient and HCT donor–derived CMV-specific T cells identified 35 days after initial infusion.

We subsequently examined the origins of the CMVpp65-specific VSTs detected after infusion (Figure 6). UPN 4417 (Figure 6A) had tetramer-binding T cells derived from third-party donors detected as late as 56 days after initial infusion of CMVpp65-VSTs. Thereafter, CMVpp65-specific T cells detected were predominantly transplant donor or host in origin, a finding correlated with the level of CD3^+^ chimerism at the time. Thus, in UPN 2386 and UPN 3907 (Figure 6, B and C), TET^+^ T cells were 100% HCT donor, while in UPN 5653 (Figure 6D), they were predominantly host in origin. In the latter case, contemporaneous cytogenetic analysis of the blood revealed full donor chimerism in myeloid cells, but 35% donor chimerism in the CD3^+^ T cells. These subjects all had sustained engraftment with full myeloid and lymphoid chimerism by 1 year after transplant. Notably, the HCT donors for UPN 2386 and UPN 3907 were CMV seronegative and had not generated detectable CMV-specific T cells during the extended period of CMV viremia preceding treatment with CMVpp65-VSTs. In both instances, HCT donor origin TET^+^ T cells were detected 21 and 56 days after infusion of third-party CMVpp65-VSTs, respectively. These data provide evidence that third-party CMVpp65-VSTs need not persist for durable responses, but may stimulate HCT donor or residual host T cells to mount a CMV-specific response that sustains control of CMV long after adoptive immunotherapy.

## Discussion

This report describes trials in which 67 allogeneic HCT recipients were treated with third-party CMVpp65-VSTs restricted by an HLA allele shared by subject and HCT donor. All were evaluable for toxicity; 59 were evaluable for response.

While subjects were eligible for treatment on trial if they had CMV viremia refractory to 2 weeks of induction antiviral therapy, most of the subjects in this cohort were treated more extensively prior to enrollment on trial and represent an especially high-risk cohort compared with the majority of those reported in the literature (5, 18, 19, 21, 23, 24). The high-risk features of the evaluable subjects in this cohort include the following: 64% were recipients of HLA nonidentical transplants; 70% received TCD grafts; 57.6% received transplants from CMV-seronegative donors; 88.1% had received 2 or more antiviral drugs; 45.7% had CMV strains genetically resistant to antiviral therapy; 67.7% had received CMV-directed therapy for more than 100 days; and 18 of 24 subjects (75%) for whom all data were available had a high cumulative load of CMV (AAUC CMV >1.5) (Table 1 and Supplemental Tables 1–3) (26). Such transplant recipients have been reported to have a significantly increased mortality due to CMV disease and associated infections (26–30).

The CMVpp65-VST infusions were well tolerated without initial clinical toxicities. However, a recurrent severe adverse event (SAE) considered possibly related was hypoxia that developed in 6 subjects. Feuchtinger et al. (32) also reported pulmonary deterioration following HCT donor–derived CMV-specific T cell infusions. These findings suggest that adoptively transferred CMV-specific T cells may traffic to sites of CMV disease and augment inflammatory responses in infected tissues, resulting in on-target toxicity. However, most subjects with CMV pneumonitis or pulmonary infiltrates at baseline did not develop this complication and responded. Thus, while the protocol consents were amended to specifically describe this risk, we did not exclude from participation patients with baseline CMV pneumonia or concern for CMV pneumonia.

Only one subject developed de novo acute GVHD, possibly related to therapy. Furthermore, we did not observe a flare or recurrence of GVHD in any of the 26 patients who had developed GVHD prior to CMVpp6VST treatment. These findings are similar to our own and others’ reported experiences with adoptive transfer of HCT donor–derived virus-specific T cells sensitized with autologous antigen-presenting cells over 3 to 5 weeks in vitro (5, 15, 17–19, 33, 34). Such T cells are extensively depleted of alloreactive T cells (Figure 2, E and F) (15, 33).

Despite their high-risk features, 38 (64.4%) of the 59 evaluable subjects achieved either CR or PR. Furthermore, both CRs and PRs were durable; only 1 responder ultimately died of CMV-related disease. Responding subjects had 6-month OS of 79%. In contrast, 8 of 21 nonresponders died of CMV, with 6-month OS of 29% (*P* < 0.001).

In univariable analyses comparing responders to nonresponders at baseline, subject characteristics that have previously been reported to be associated with a high risk of CMV mortality (26, 31), namely recipients having TCD grafts and failing to achieve a CD4^+^ T cell count of 50/μL or more, were significantly (*P* = 0.007) associated with failure to respond to CMVpp65-VSTs. In addition, levels of CD3^+^, CD4^+^, and CD8^+^ at baseline were significantly (*P* = 0.003, *P* = 0.004, and *P* = 0.005 respectively) lower in nonresponders. We and others (27, 29) have reported a higher incidence of CMV infections in recipients of in vitro TCD allografts, potentially reflecting the markedly lower number of T cells and particularly CMV-specific T cells from a seropositive donor that would be transferred in a TCD graft compared with an unmodified transplant. T cell depletion can also delay recovery of functional CMV-specific T cells, since such recovery depends more on the maturation of donor-derived lymphoid precursors from the graft within the host thymus than on the peripheral expansion of mature T cells that are present in an unmodified graft. This is particularly the case for older subjects whose T cell production is more limited due to thymic atrophy (35, 36). However, in our cohort, although nonresponders were somewhat older, age did not significantly affect response. In addition, the response rate for recipients of transplants from CMV-seropositive donors did not differ significantly from the response rate for recipients of transplants from seronegative donors. These findings likely reflect the preponderance of TCD graft recipients in our study, since T cell depletion markedly reduces the number of CMV-reactive T cells transferred in the graft.

Irrespective of the type of transplant, we observed a significantly lower response rate among subjects with lower levels of both CD4^+^ and CD8^+^ T cells at baseline prior to infusions of third-party CMVpp65-VSTs. Furthermore, while the CMVpp65-VSTs expanded well in recipients of TCD grafts and induced responses in 56% of cases, the levels of circulating CMV-specific IFN-γ^+^ T cells achieved and maintained in responding subjects were lower than those detected in recipients of unmodified transplants.

Unexpectedly, we also found that the degree of expansion of IFN-γ^+^ and TET^+^ CMVpp65-specific T cells in responders and nonresponders did not differ significantly. This observation contrasts with the differentially higher levels of virus-specific T cells detected in patients responding to treatment with HCT donor–derived CMV-specific T cells that can also engraft after transfer (14). There are likely multiple parameters important in identifying peripheral blood correlates of response to third-party VSTs, including the virus being treated, the recipient immune milieu (e.g. baseline greater than 50 CD4^+^ T cells/μL and TCD HCT), the donor/host origin of the expanded population identified (as demonstrated in Figure 6), and the functional phenotype of these expanding T cells (as suggested by other authors; refs. 37–39). Technical limitations prevented us from analyzing these independently, so there may be critical differences in responders and nonresponders that we were unable to detect. Specifically, we previously demonstrated (40) a difference in peak EBV-CTLp expansion in recipients of third-party EBV-VSTs responding versus not responding to therapy, while some authors have demonstrated clinical responses in the absence of detectable peripheral expansion (37).

Our findings may thus reflect variable limitations to the antiviral activity of third-party CMVpp65-VSTs against viral epitopes presented by infected host cells or to their capacity to recruit endogenous HCT donor–derived or residual host T cells. This would be expected to affect responses, especially if effective clearance by T cells specific for certain epitopes depends upon attainment and/or maintenance of critical levels of these T cells both in sites of infection and the circulation. A recent prospective study (41) employed dextramers binding CMV epitopes complexed with their presenting HLA alleles to quantitate reconstitution of CMV-specific T cells and identified levels of over 0.5 CMV-specific CD8^+^ T cells/μL by day 45 after transplant as protective from clinically relevant CMV reactivation. Our cohort was too small to identify a range of CMV-specific T cell levels associated with clearance of CMV viremia. However, such quantitations of CMV-specific T cells in larger prospective trials should help discern other T cell or host characteristics influencing responses to CMV-VSTs.

Taken together, these data coupled with our findings regarding the origins of CMVpp65 TET^+^ T cells following the first 2 cycles of CMVpp65CTL infusions indicate that CMVpp65-specific T cells of HCT donor origin may contribute to the high clinical response rates to adoptive therapy that we and others have observed in recipients of unmodified grafts (5, 18, 19, 24).

Notably, several high-risk features of CMV infection and the virus causing it did not significantly affect responses to CMVpp65-VSTs. For example, subjects with invasive disease, including subjects with meningoencephalitis or chorioretinitis, and subjects infected with mutant CMV strains resistant to antiviral drugs fared as well as those without. Furthermore, the number of different antivirals that the subject had received did not distinguish responders from nonresponders. However, our finding that concurrent treatment with ganciclovir is associated with a lower response rate raises concerns that the reported immunosuppressive effects of this antiviral (7, 42, 43) may also affect adoptively transferred virus-specific T cells that are predominantly of a TEM or TEMRA phenotype. This finding contrasts with the lack of inhibitory effects of cyclosporine, tacrolimus, or sirolimus on the function or expansion of CMVpp65-VSTs in this trial or other trials of virus-specific T cells observed after adoptive transfer into transplant recipients concurrently receiving these agents for GVHD prophylaxis. These subjects respond well to such virus-specific T cells, likely reflecting the limited activity of the immunosuppressive drugs against memory T cells (44–46).

This trial of third-party–derived CMVpp65-VSTs is, to our knowledge, the first to identify both the CMVpp65 peptide epitope targeted by each virus-specific T cell line infused and the epitope’s presenting HLA allele. Despite the diversity of our transplant subjects and their mostly unrelated donors, selection of third-party CMVpp65-VSTs restricted by an HLA allele shared by the subject and transplant donor required the use of CMVpp65-VSTs restricted by only 25 prevalent HLA alleles that were specific for 19 different CMVpp65 peptide epitopes. Because of the critical importance of CD8^+^ CMVpp65-VSTs T cells to effective immunity, we preferentially selected CD8^+^ CMVpp65-VSTs. These were specific for 1 to 3 epitopes presented by HLA-A or HLA-B alleles typed at high resolution. Only 2 subjects required selection of CD4^+^ T cell lines specific for epitopes presented by a class II HLA allele. Overall, responses among groups of subjects treated with CMVpp65-VSTs restricted by the same HLA allele were similar to the 64% response rate for the entire group. Notably, although CMVpp65-VSTs specific for the epitopes presented by HLA B0702 and by HLA A0201 are consistently immunodominant in individuals inheriting these alleles and have been hypothesized to provide particularly effective resistance, response rates to CMVpp65-VSTs restricted by these alleles were not different from responses to CMVpp65-VSTs restricted by other alleles.

Conversely, the consistent failure (0/7) of subjects treated with CMVpp65-VSTs restricted by allelic variants of HLA B35 to respond was highly significant (*P* = <0.001). Previously, inheritance of B35 has been associated with rapid progression of HIV (47–49) postulated to be due to the reduced antiviral activity of T cells specific for particular epitopes presented by HLA B35 alleles (50, 51). However, the mechanisms contributing to the altered activity observed are not well defined. In a separate report describing the specificities and function of CMVpp65-VSTs in our bank, Hasan et al. provide evidence of HLA-specific impairments of CMVpp65 epitope presentation by HLA B35 alleles in CMV-infected cells (52).

Although the higher long-term survival in subjects achieving a CR or PR following CMVpp65CTL infusions is clinically meaningful, the basis for the durability of responses, particularly PRs, remains uncertain. At issue is the following: how is a PR sustained, since third-party CMVpp65-VSTs would be expected to be rejected relatively early after infusion? Existing data from our own and other trials are extremely limited. However, third-party CMV-specific T cells were not detected in our trials beyond 8 weeks and in other reports no later than 12 weeks after initial infusion (5, 18). The durability of PRs observed in our cohort may, in part, reflect the more stringent criteria we used to define a PR (i.e., 2log_10_ reduction of CMV DNA versus a 50% reduction in other reported studies; refs. 5, 18, 19). Indeed, in our cohort, 6 of the subjects that achieved a PR maintained the same low level of viremia achieved after CMVpp65CTL infusions for 6 months without additional antiviral treatment. Maintenance of such low levels suggests host control of the virus.

Our group previously correlated a milestone of immune reconstitution, namely a level of CD4^+^ T cells of 50 × 10^6^/L or more in the blood, at baseline, with long-term responses to adoptive therapy with HCT donor– or third-party–derived CMVpp65-VSTs. Extending beyond this validated milestone, it is notable that in the current study, higher numbers of both the CD3^+^CD4^+^ (*P* = 0.004) and the CD3^+^CD8^+^ (*P* = 0.005) T cells in the blood at baseline were significantly associated with response. Furthermore, while third-party TET^+^ CMVpp65-VSTs could be detected by short tandem repeat (STR) analysis up to day 56, most of the CMVpp56 peptide–responsive IFN-γ^+^CD3^+^ T cells and CMVpp65 peptide/HLA tetramer^+^CD8^+^ T cells detected in the subjects as early as day 35 and all those detected after day 56 were of either transplant donor or patient origin. Strikingly, this includes identification of CMVpp65-specific T cells of HCT donor origin in subjects in whom the HCT donor was CMV seronegative and had no evidence of CMV-specific T cells prior to infusion of third-party CMVpp65-VSTs.

These data thus provide direct evidence that, although initial expansions of the adoptively transferred CMVpp65-VSTs are closely associated with and likely initiate the responses that lead to clearance of CMV disease and viremia, durability of responses, particularly the PRs, is mediated by recruitment of engrafted donor or residual host CMV-specific T cells, including CD8^+^ T cells specific for CMVpp65 epitopes presented by the same HLA alleles as those by which the third-party T cells were restricted. Such recruitment has previously been hypothesized to contribute to long-term responses to transplant donor–derived CMV-CTLs and also invoked to explain persistence of responses to third-party virus-specific T cells (19). It is also consistent with a growing body of literature suggesting broadening of immune responses even after infusion of autologous antigen-specific T cells (53, 54).

Limitations of our study relate to inclusion of subjects referred to our center from outside sites after transplant. This limited our assessments of overall viral load, resistance to antivirals, and long-term immune responses to CMV. Additionally, this manuscript reports on subjects treated prior to wide-scale use of letermovir prophylaxis. However, although letermovir prophylaxis has decreased the incidence of CMV reactivation and infection, a proportion of patients either fail prophylaxis or develop serious CMV infection after letermovir is discontinued. The subjects treated in this cohort were at high risk of death from CMV and had failed prolonged therapy with ganciclovir, foscarnet, or cidofovir, representing a group for whom novel therapeutic approaches are still needed.

In conclusion, adoptive transfer of well-characterized third-party–derived CMVpp65-VSTs sensitized in vitro to 15-mer peptides spanning CMVpp65 and selected based on matching for 2 or more HLA alleles and restriction by an HLA allele shared by the subject and HCT donor is associated with a tolerable toxicity profile and strikingly durable responses in 64% of recipients. These third-party CMVpp65 CTLs can clear CMV infections and provide a survival benefit to responding subjects who have failed prolonged therapy with antiviral drugs and have multiple risk factors for treatment failure.

## Methods

### Clinical trial.

Our phase I and phase II trials were designed to assess the toxicity and antiviral activity of CMVpp65-VSTs generated from healthy CMV-seropositive donors other than the subject’s transplant donor. CTL lines were selected from a bank of 138 cryopreserved CMVpp65CTL lines based on matching for 2 or more HLA alleles of the subject and restriction by an HLA allele shared by the subject and the subject’s HCT donor. The bank of CMVpp65-VSTs was established from HCT donors who specifically consented to use of their T cells for individuals other than the recipient of their HCT donation.

### Subjects.

Eligible subjects were allogeneic HCT recipients of any age who had either clinical CMV disease or CMV viremia that either progressed during treatment or persisted despite treatment for 2 or more weeks with induction doses of antiviral drugs. Subjects were identified by their treating attending or referring center and were screened for availability of an appropriate third-party CMVpp65-VST line prior to signing treatment consent. Demographics related to race and ethnicity were based on participant self-reporting. Between July 2009 and July 2017, 71 subjects were enrolled on 3 successive trials, including phase I/II trials amended to include third-party CMVpp65CTL (ClinicalTrials.gov NCT00674648, NCT01646645) and a subsequent phase II trial specific for third-party CMVpp65-VSTs (ClinicalTrials.gov NCT02136797). Subjects who were moribund, pregnant, requiring vasopressor support, or receiving doses of 0.5 mg/kg or more prednisone or its equivalent or (in NCT02136797) extracorporeal photopheresis or methotrexate for treatment of GVHD were ineligible.

Based on our trial of transplant donor–derived CMVpp65-VSTs (15), subjects received infusion in 35-day cycles. Cycles started with the first of 3 weekly infusions of 1 × 10^6^ third-party CMVpp65-VSTs/kg/dose. Recipients were evaluated for initial response on day 35 (±7 days) following the first infusion and followed on trial for 6 months after the last infusion and for survival for 2 years. Endpoints included incidence of severe toxicities or acute GVHD, clinical and virological responses, and alterations in CMVpp65-specific T cells detected after infusion. In this analysis, CR is defined as complete clearance of viremia and biopsy-proven resolution of invasive disease; PR is defined as a 2log_10_ decrease in CMV viral load and resolution of clinical symptoms related to disease. Subjects without toxicity who did not achieve CR following the initial course of CMVpp65 could receive additional 5-week cycles of CMVpp65-VSTs from the same or a different donor.

### Generation of CMVpp65 CTLs.

We generated CMVpp65-VSTs as previously described (55, 56). Briefly, CD3^+^-enriched T cell fractions, isolated from PBMCs by depletion of adherent monocytes and immunoadsorption of NK cells, were stimulated with irradiated autologous CAMS or EBV-BLCLs (generated as previously described; refs. 4, 15, 56, 57) loaded with a pool of overlapping pentadecapeptides spanning the sequence of CMVpp65 (Invitrogen) and propagated in vitro with weekly restimulations, and supplementation with IL-2 beginning at day 10 to 16 (4, 55). After 28 days, T cells were harvested, counted, and tested for CMVpp65-specific cytotoxicity and lack of alloreactivity (4, 55, 57), microbiological sterility, and endotoxin levels. CMVpp65-VSTs meeting release criteria were cryopreserved in calculated doses for subsequent administration.

### Characterization of CMVpp65CTL lines.

The CD3^+^, CD4^+^, and CD8^+^ T cells, CD3^–^CD56^+^ NK cells, and CD20^+^ B cells in CMVpp65-VSTs were quantitated by FACS analysis. A subset of CMVpp65CTL lines were further assessed for characterization of TEMRA, TEM, and TCM using antibodies to CD62L, CCR7, and CD45RA.

The monoclonal antibodies used for the FACS analyses included antibodies to the following: CD3 BV786 (catalog 563800), CD2OPE (catalog 346581), CD45RAPE (catalog 555489), CD45ROFITC (catalog 555492), CD45 RO APC (catalog 559865), and CD62L FITC (catalog 555543) from BD Biosciences; CD4 APC Cy 7 (catalog 300518), CD8 BV 421 (catalog 301036), CD45 RO PerCP Cy5.5 (catalog 304026), CD62L PE Cy 7 (catalog 304822), TNFa APC (catalog 502912), and CCR7 BV605 (catalog 353214) from BioLegend; and CD56 APC (catalog 130-113-30J) and IFN-γ FITC (catalog 130-091-091-641) from Miltenyi Biotec.

We measured specific cytotoxic activity of CMVpp65-VSTs against CMVpp65 peptide–loaded targets using a standard ^51^chromium release assay (57). In addition, CD8^+^ and/or CD4^+^ CMVpp65-specific T cells producing IFN-γ and TNF-α in response to CMVpp65 peptides were quantitated, using a modification of the technique of Waldrop et al. (58) previously described (4, 55, 57).

The CMVpp65 epitope specificities of each CMVpp65CTL line were identified using a mapping grid of CMVpp65 peptide subpools as previously described (4, 55). T cell responses to specific peptides or subpools of CMVpp65 were quantitated by measuring the number of IFN-γ^+^ T cells generated upon secondary stimulation with autologous peptide–loaded CAMs (4, 55). To define HLA restrictions of epitope-specific CMVpp65-VSTs, their cytotoxic activity was measured against a panel of PHA blasts either unloaded or loaded with the recognized peptide, each sharing a single HLA allele with the donor of the T cell line, as previously described (4, 57). For a subset of CMVpp65-VSTs of defined epitope specificity and HLA restriction, we quantitated T cells binding CMVpp65 epitope/HLA tetramers (e.g., NLVPMVATV/HLA-A*0201, QYDPPVAALF/HLA-A*2402,and TPRVTGGGAM/HLA-B*0702 tetramers) (Beckman-Coulter) as previously described (56).

### Monitoring of subjects after CMVpp65CTL infusions.

Subjects were monitored for toxicity for 30 days and GVHD for 100 days after the last infusion. Toxicities were graded using the Common Terminology Criteria for Adverse Events (CTCAE) (version 4, 2009). Acute GVHD was graded by IBMTR Consensus criteria (59). CMV responses to CMVpp65CTL infusions were assessed at 35 (±7) days after the first infusion of each cycle and for 6 months fter the last infusion of CMVpp65-VSTs. Responses were based on clinical, radiologic, and biochemical alterations of affected organs and/or alterations of viral load in blood or affected tissues.

CMV DNA copy numbers in the blood, measured by quantitative real-time PCR assay (60, 61) were monitored prior to T cell infusions at weekly intervals for 6 weeks and at least monthly for 6 months thereafter to ascertain continued response or progression. Death was attributed to CMV when caused by progressive functional deterioration of an organ infected with CMV or toxicity related to treatment of CMV (62). T cell responses were measured in sequential blood samples from treated subjects by quantitating IFN-γ^+^ T cells responding to the pool of CMVpp65 peptides by FACS as previously described (4). In recipients of CMVpp65-VSTs restricted by certain HLA alleles, responses were measured by quantitating CMV peptide/HLA tetramer^+^ T cells by FACS (56).

When sufficient numbers of CMVpp65-specific T cells could be isolated from PBMC samples after infusion by FACS sorting of CMVpp65 peptide/HLA tetramer^+^ T cells (*n* = 3) or by isolating CMVpp65 peptide–reactive IFN-γ^+^ T cells using the IFN-capture technique (*n* = 5), they were analyzed for genetic origin by STR analysis as previously described (40, 63).

### Statistics.

All analyses were completed using the statistical software R, version 4.1.0. Subject and CMVpp65CTL characteristics were compared between responders and nonresponders using χ^2^ tests, Fisher’s exact test, and Wilcoxon’s rank-sum tests. Survival curves were generated from the time of response to death using the Kaplan-Meier method and compared using log-rank tests. Cumulative incidences of CMV mortality (with death due to other causes as competing events) were estimated using an Aalen-Johansen estimator and compared between response groups using Gray’s test. A *P* value of 0.05 was used to determine statistical significance in the analysis.

### Study approval.

The trials were approved by the Institutional Review Board at Memorial Sloan-Kettering Cancer Center, the National Marrow Donor Program, and the FDA. All participants provided written, informed consent.

### Data availability.

Deidentified participant data will be made available from the corresponding author upon request and signature of data transfer agreement.

## Author contributions

All authors have critically reviewed the manuscript for intellectual content, accuracy, and integrity and have given final approval of the version submitted for publication. SEP and RJO wrote and ran the protocol, accrued and treated subjects, participated in line selection, analyzed and interpreted the data, and drafted the manuscript. AH, KP, TV, and AS performed analyses of T cell lines and treated subjects. AH also selected lines and treated subjects. ED generated T cell lines, performed analyses of T cell lines, and participated in line selection. PBD participated in line selection and accrued and treated subjects on trial. IRS, MC, AM, GAP, and YS provided analysis and interpretation of the data. JY and MA performed analysis of chimerism by STR. FB, HCM, CC, KJC, SG, NAK, GK, AJ, EP, MAP, IP, CSS, RT, and JWY accrued and treated subjects.

## Supplementary Material

Supplemental data

Trial reporting checklists

ICMJE disclosure forms

## Figures and Tables

**Table 7:**
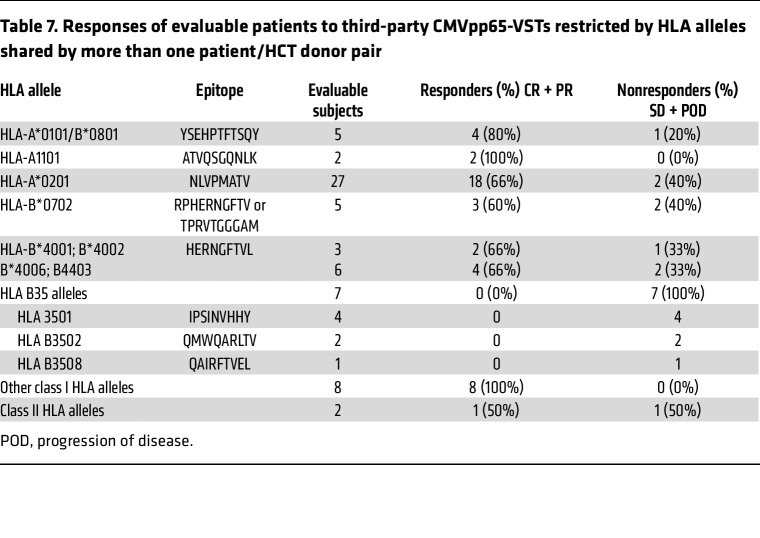
Responses of evaluable patients to third-party CMVpp65-VSTs restricted by HLA alleles shared by more than one patient/HCT donor pair

**Table 6:**
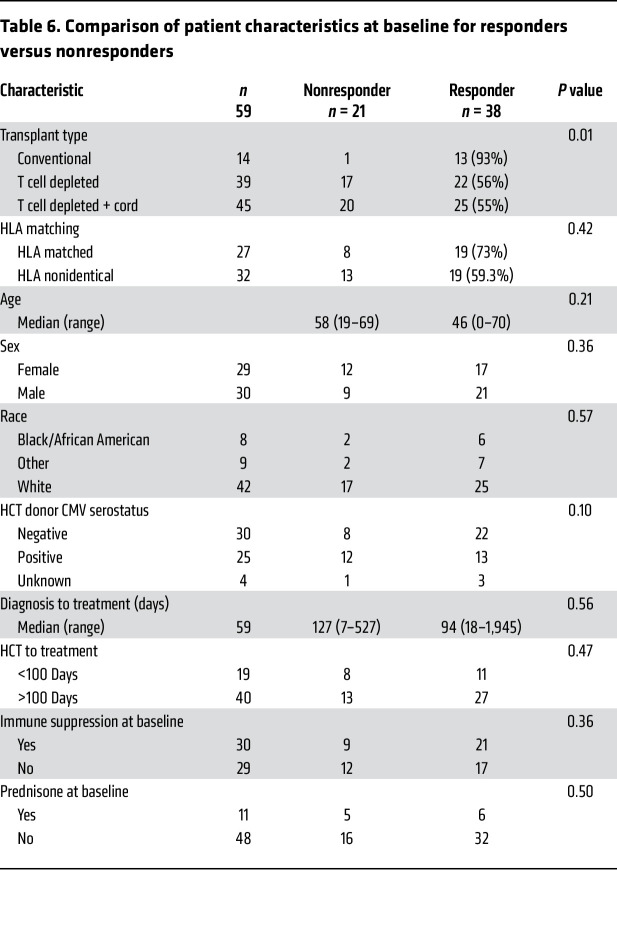
Comparison of patient characteristics at baseline for responders versus nonresponders

**Table 5:**
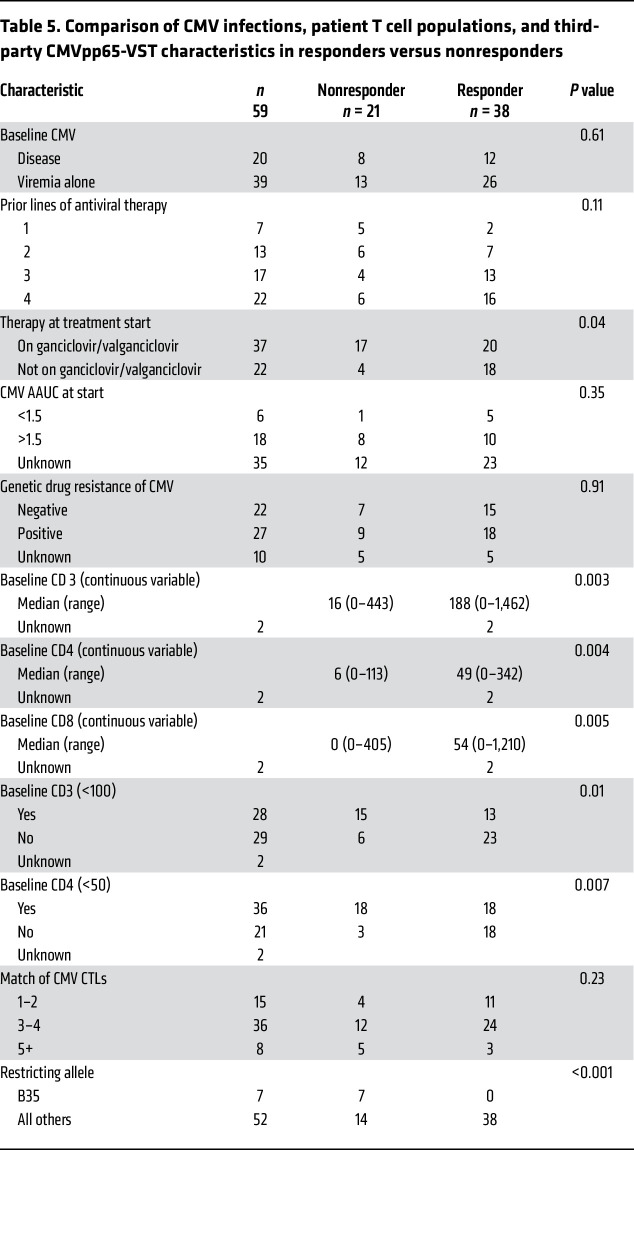
Comparison of CMV infections, patient T cell populations, and third-party CMVpp65-VST characteristics in responders versus nonresponders

**Table 4:**
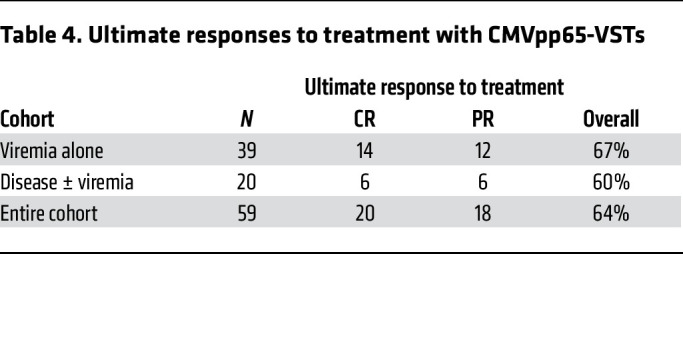
Ultimate responses to treatment with CMVpp65-VSTs

**Table 3:**
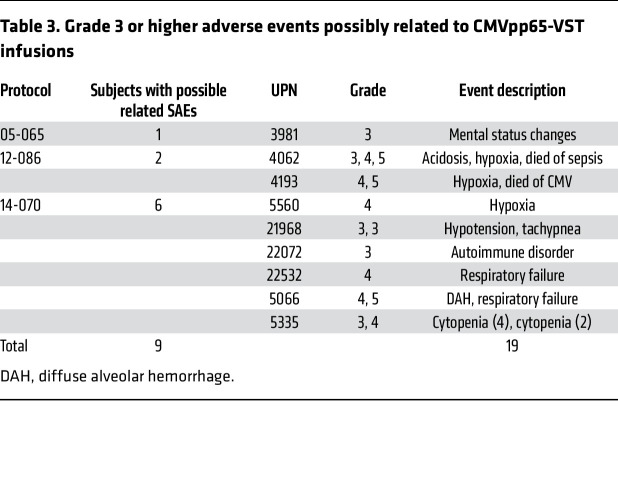
Grade 3 or higher adverse events possibly related to CMVpp65-VST infusions

**Table 2:**
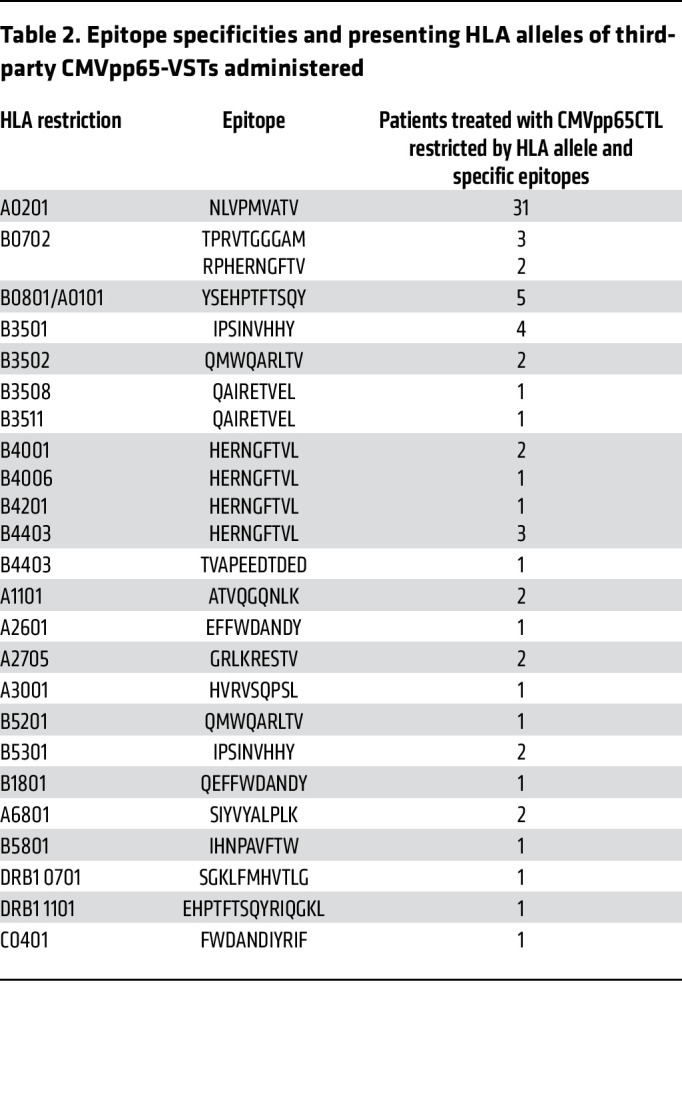
Epitope specificities and presenting HLA alleles of third-party CMVpp65-VSTs administered

**Table 1:**
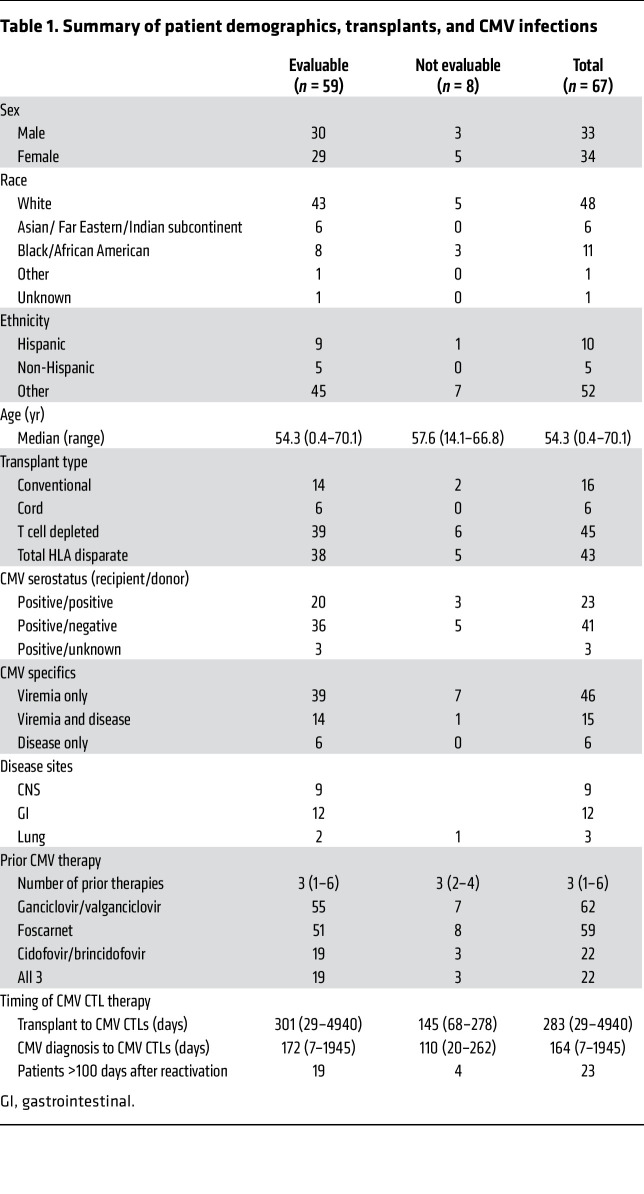
Summary of patient demographics, transplants, and CMV infections

## Version 1 03/23/2023

In-Press Preview

## Version 2 05/15/2023

Electronic publication
