# Supplementary material for: Third-party cytomegalovirus-specific T cells improved survival in refractory cytomegalovirus viremia after hematopoietic transplant
Source: J Clin Invest. 2023 May 15;133(10):e165476. doi: 10.1172/JCI165476 (PMC10178844; doi:10.1172/JCI165476)
Supplement: Supplemental data [file jci-133-165476-s120.pdf]

Supplemental Table 1. Pre-Treatment Characteristics of Treated Patients

UPN	Age At Start Date	Sex	Diagnosis	Source	Donor	Mismatch	Prior aGvHD	Grade	Immune Supression at Baseline	CNI	Siro	MMF	BUD	HC	MP	Pred	Other	Pred Equiv	Responder/Non	baseline CD4<50 (yes/no)	baseline CD4<200 (yes/no)	Pre-infusion CD4	Pre-infusion CD8	Risk Factors
5554	13.7	M	SSD	cBM	MRD	N/A	Yes	3	Yes	Yes	No	No	No	No	No	No	No	-	RESPONDER	no	yes	70	141	4
5558	28.5	M	SAA	cBM	mMUD	N/A	No	N/A	Yes	Yes	No	No	No	No	No	No	No	-	RESPONDER	yes	yes	17	11	3
5559	70.1	F	AML	cBM	mMRD	5/10	No	1	Yes	Yes	No	No	No	No	No	No	No	-	RESPONDER	no	yes	150	285	5
21856	10.4	F	SAA	cBM	MUD	N/A	No	N/A	Yes	Yes	No	No	No	No	No	No	No	-	RESPONDER	n/a	n/a	n/a	n/a	4
21968	0.4	F	NMD	cBM	MUD	10/10	No	N/A	Yes	Yes	No	Yes	No	No	No	No	No		RESPONDER	yes	yes	41	243	2
22156	43.6	M	SAA	cBM	mMUD	5/10	No	N/A	Yes	Yes	No	Yes	No	No	No	Yes	No	0.047	RESPONDER	no	yes	57	57	6
3802	52.1	F	NHL	cPBSC	MRD	N/A	Yes	2	Yes	Yes	No	No	No	No	No	Yes	No	0.2	N/E	yes	yes	15	11	5
5329	66.8	F	ALL	cPBSC	MUD	N/A	No	N/A	No	No	No	No	No	No	No	No	No		N/E	no	yes	63	450	3
5462	66.3	F	NHL	cPBSC	MUD	N/A	Yes	2	Yes	Yes	No	No	No	No	Yes	No	NO	0.027	RESPONDER	no	no	246	881	6
5551	29.8	M	NMD	cPBSC	MRD	N/A	No	1	No	No	No	No	No	No	No	No	No	-	RESPONDER	no	yes	151	529	3
5552	26.8	M	ALL	cPBSC	MUD	N/A	Yes	4	Yes	No	No	No	No	No	No	No	No	-	RESPONDER	no	yes	148	251	5
5557	49.4	F	ALL	cPBSC	mMUD	9/10 (a)	No	N/A	No	No	No	No	No	No	No	No	No	-	RESPONDER	no	yes	92	141	4
5560	63.1	F	NHL	cPBSC	MUD	N/A	Yes	2	No	No	No	No	No	No	No	No	No	-	NON RESPONDER	yes	yes	0	0	7
5561	55.2	M	NHL	cPBSC	mMUD	8/10 (DR, DQ)	No	1	Yes	No	Yes	No	No	No	No	No	No	-	RESPONDER	no	no	202	1210	5
22230	16.3	M	NMD	cPBSC	mMUD	9/10	Yes	2	Yes	No	No	No	No	Yes	No	No	No	0.11	RESPONDER	yes	yes	0	0	7
22323	63.7	M	ALL	cPBSC	MRD	N/A	Yes	2	Yes	No	Yes	No	Yes	Yes	No	No	No	0.16	RESPONDER	no	yes	113	176	4
2386	54.3	F	NHL	TCD	MRD	N/A	Yes	3	Yes	Yes	No	No	No	No	No	Yes	No	0.036	RESPONDER	no	no	286	321	5
3619	14.4	M	ALL	TCD	mMUD	9/10 (A)	No	N/A	YEs	No	No	No	No	Yes	Yes	No	No	0.5	N/E	yes	yes	0	0	6
3907	16.2	M	AML	TCD	mMUD	9/10 (A)	Yes	2	Yes	No	No	No	No	No	No	Yes	No	0.18	RESPONDER	no	no	237	30	8
3951	23.5	F	AML	TCD	mMUD	9/10 (A)	Yes	3	Yes	No	No	No	No	No	No	Yes	No	0.03	RESPONDER	yes	yes	15	120	8
3972	36.6	F	HLH	TCD	mMUD	8/10 (DR/DQ)	Yes	3	Yes	Yes	No	No	No	No	Yes	No	No	0.2	NON RESPONDER	yes	yes	10	45	10
3981	66.1	M	MDS	TCD	mMUD	9/10 C	No	N/A	Yes	No	No	No	No	Yes	No	No	No	0.08	NON RESPONDER	yes	yes	0	0	9
4062	68.6	F	AML	TCD	MRD	N/A	No	N/A	No	No	No	No	No	No	No	No	No	-	RESPONDER	no	yes	128	36	4
4124	14.1	F	NMD	TCD	mMUD	9/10 (C)	No	N/A	No	No	No	No	No	No	No	No			N/E	yes	yes	12	0	6
4232	64.8	M	MM	TCD	mMUD	9/10 A	No	N/A	No	No	No	No	No	No	No	No	No	-	NON RESPONDER	yes	yes	0	0	6
4234	65.4	F	MDS	TCD	MUD	N/A	No	N/A	No	No	No	No	No	No	No	No	No		N/E	yes	yes	11	155	2
4247	69.2	M	AML	TCD	MRD	N/A	No	N/A	No	No	No	No	No	No	No	No	No	-	NON RESPONDER	yes	yes	0	0	4
4268	62.1	F	ALL	TCD	MUD	N/A	No	2	No	No	No	No	No	No	No	No	No	-	RESPONDER	yes	yes	0	0	5
4286	20.3	F	AML	TCD	mMUD	9/10	No	N/A	Yes	Yes	No	No	No	No	No	Yes	No	0.093	NON RESPONDER	yes	yes	0	0	5
4417	61.5	M	MM	TCD	mMUD	9/10 (DR)	No	N/A	No	No	No	No	No	No	No	No	No	-	RESPONDER	no	yes	91	455	5
4508	18.8	F	ALL	TCD	mMUD	9/10 (A)	No	N/A	No	No	No	No	No	No	No	No	No	-	NON RESPONDER	yes	yes	0	0	4
4518	68.9	M	MDS	TCD	MUD	N/A	Yes	2	No	No	No	No	No	No	No	No	No	-	RESPONDER	no	yes	135	34	3
5062	51.6	F	AML	TCD	MUD	N/A	No	N/A	No	No	No	No	No	No	No	No	No	-	NON RESPONDER	no	yes	56	67	4
5066	63.2	M	MM	TCD	mMUD	9/10 (A)	Yes	2	Yes	No	No	No	Yes	No	No	No	No		N/E	no	yes	196	279	6
5073	69.5	M	AML	TCD	mMUD	8/10 DR/DQ	Yes	2	No	No	No	No	No	No	No	No	No	-	RESPONDER	yes	yes	0	0	6
5089	15.5	M	AML	TCD	MRD	N/A	No	N/A	No	No	No	No	No	No	No	No	No	-	RESPONDER	yes	yes	33	491	4
5100	60.2	F	MM	TCD	MRD	N/A	No	N/A	No	No	No	No	No	No	No	No	No	-	NON RESPONDER	yes	yes	0	0	3
5208	64.9	F	AML	TCD	mMUD	9/10 (B)	No	N/A	No	No	No	No	No	No	No	No	No		N/E	no	yes	99	53	6
5265	21.2	F	MDS	TCD	mMUD	8/10 (AC)	No	N/A	No	No	No	No	No	No	No	No	No	-	NON RESPONDER	yes	yes	0	0	4
5335	16.7	M	ALL	TCD	mMUD	8/10, 7/8 B/DQ	Yes	2	No	No	No	No	No	No	No	No	No		N/E	yes	yes	4	8	5
5337	66.2	M	MDS	TCD	MUD	N/A	No	N/A	No	No	No	No	No	No	No	No	No	-	NON RESPONDER	yes	yes	16	60	3
5526	66.9	M	MDS	TCD	MUD	N/A	No	1	No	No	No	No	No	No	No	No	NO		RESPONDER	yes	yes	29	88	7
5550	7.4	M	ALL	TCD	mMRD	5/10	No	1	Yes	Yes	No	No	No	No	Yes	No	No	0.299	RESPONDER	no	yes	136	408	7
5562	14.3	F	NMD	TCD	mMUD	7/10 (A/DR/DQ)	No	N/A	No	No	No	No	No	No	No	No	No	-	RESPONDER	no	yes	103	16	3
5653	68.2	F	AML	TCD	MRD	N/A	No	N/A	yes	No	No	No	Yes	No	No	No	No	-	RESPONDER	yes	yes	9	28	4
5821	52.9	F	AML	TCD	mMRD	5/10	Yes	2	YEs	No	No	Yes	Yes	Yes	No	Yes	No	0.13	NON RESPONDER	yes	yes	20	38	9
5841	19.7	F	CML	TCD	mMUD	8/10	Yes	2	Yes	Yes	No	No	Yes	No	Yes	No	No	0.07	RESPONDER	yes	yes	0	50	7
5842	13.9	M	AML	TCD	mMRD	5/10	No	N/A	No	No	No	No	No	No	No	No	No	-	RESPONDER	yes	yes	7	1	4
5873	57.6	M	ALL	TCD	MRD	N/A	Yes	2	No	No	No	No	No	No	No	No	No	-	RESPONDER	yes	yes	0	7	5
6048	57.7	F	AML	TCD	mMRD	5/10	yes	4	Yes	No	No	Yes	No	No	Yes	Yes	No	0.09	NON RESPONDER	yes	yes	37	405	7
6049	59.1	M	PTCL	TCD	MUD	N/A	No	N/A	No	No	No	No	No	No	No	No	No	-	NON RESPONDER	yes	yes	32	35	6
6098	67.9	F	MPD	TCD	mMRD	8/10, (DR/DQ)	No	N/A	No	No	No	No	No	No	No	No	No		NON RESPONDER	yes	yes	0	0	4
6106	1.3	M	ALL	TCD	mMUD	9/10	No	N/A	No	No	No	No	No	No	No	No	No	-	RESPONDER	yes	yes	3	40	6
6115	54.7	F	MM	TCD	mMUD	9/10	Yes	3	Yes	No	No	No	Yes	No	No	Yes	No	-	RESPONDER	yes	yes	0	0	6
21890	55.2	F	MM	TCD	MUD	N/A	No	N/A	No	No	No	No	No	No	No	No	No	-	NON RESPONDER	yes	yes	8	92	6
22022	60.4	F	AML	TCD	MUD	10/10	Yes	2	Yes	No	No	No	Yes	No	No	No	No		RESPONDER	yes	yes	26	132	9
22072	39.3	M	ALL	TCD	MRD	10/10	No	N/A	No	No	No	No	No	No	No	No	No		RESPONDER	yes	yes	0	0	4
22174	1	M	NMD	TCD	mMUD	9/10	No	N/A	No	No	No	No	No	No	No	No	No	-	RESPONDER	yes	yes	2	30	5
22269	31	M	ALL	TCD	MUD	N/A	No	1	No	No	No	No	No	No	No	No	No	-	NON RESPONDER	yes	yes	0	0	6
22333	42.2	F	AML	TCD	MRD	N/A	No	N/A	No	No	No	No	No	No	No	No	No	-	RESPONDER	yes	yes	0	0	4
22532	21	M	NMD	TCD	mMUD	8/10'DR/DQ	No	N/A	Yes	No	No	No	Yes	No	Yes	No	No	0.2	NON RESPONDER	no	yes	113	13	5
4101	60.5	F	AML	UCB	mMUD	5/6, 4/6	Yes	2	Yes	No	No	Yes	Yes	No	No	No	No	-	RESPONDER	no	no	214	13	4
4193	59	M	ALL	UCB	mMUD	DUCB Haplo	Yes	2	Yes	Yes	No	No	No	No	No	No	No	-	NON RESPONDER	yes	yes	8	0	6
5146	45.9	F	CML	UCB	mMUD	dCB/haplo	Yes	4	Yes	Yes	No	Yes	No	No	No	Yes	No	0.299	NON RESPONDER	no	yes	82	23	6
5553	62.2	M	CML	UCB	mMUD	3/6 and 4/6	No	N/A	No	No	No	No	No	No	No	No	No	-	RESPONDER	n/a	n/a	n/a	n/a	6
5555	65	M	CML	UCB	mMUD	6/8, 5/8	Yes	3	Yes	Yes	No	No	No	No	No	Yes	hotopheres	0.03	NON RESPONDER	yes	yes	6	130	7
6072	60.7	F	AML	UCB	mMUD	4/6, 4/6	Yes	2	Yes	Yes	No	No	Yes	No	No	No	No		RESPONDER	no	no	342	902	7

Supplemental Table 2. Peri-Treatment Characteristics of Treated Patients

UPN	CMV Donor Serostatus	HCT to CMV CTLs (Days)	HCT to CMV CTLs < vs > 100 days	CMV to CMV CTLs	CMV to CMV-CTLs	Type	Disease	Chest X-ray at baseline	Prior FOS	Prior G/VAL	Prior CID/CMX	Prior	Resistance	Resistance To Ganciclovir	Resistance To Foscarnet	Resistance To Cidofovir	# Resistance	CON FOS	CON G/VALG	CON CID/CMX	#	Allele Match	Restriction	Best response	Responder/Non	Survival Status
5554	POSITIVE	128	>100	109	>100	Viremia	NA	ABNORMAL	Yes	Yes	Yes	4	YES	Yes	No	No	1	No	No	Yes	1	4	B0801	CR	RESPONDER	Alive
5558	NEGATIVE	119	>100	51	<100	Viremia	NA	NORMAL	Yes	Yes	No	3	N/A	-	-	-	0	No	No	No	0	4	A 0101, B0801	CR	RESPONDER	Alive
5559	NEGATIVE	148	>100	117	>100	Viremia	NA	ABNORMAL	Yes	Yes	Yes	5	YES	Yes	Yes	Yes	3	Yes	Yes	No	3	1	B1801	PR	RESPONDER	Died of other causes
21856	NEGATIVE	129	>100	80	<100	Both	lung	ABNORMAL	Yes	Yes	No	4	YES	Yes	No	Yes	2	Yes	No	No	2	3	B2705, C0202	PR	RESPONDER	Alive
21968	NEGATIVE	29	≤100	67	<100	Viremia	NA	NORMAL	Yes	Yes	No	3	NO	No	No	No	0	No	Yes	No	2	1	A0101	CR	RESPONDER	Alive
22156	NEGATIVE	122	>100	92	<100	Viremia	NA	NORMAL	yes	Yes	Yes	3	YES	Yes	No	No	1	Yes	Yes	Yes	3	4	A0201	PR	RESPONDER	Alive
3802	NEGATIVE	181	>100	82	<100	Viremia	NA	ABNORMAL	Yes	Yes	Yes	3	N/A	-	-	-	0	No	Yes	No	1	3	A0201	SD*	N/E	Died of other causes
5329	NEGATIVE	92	≤100	59	<100	Viremia	NA	NORMAL	Yes	Yes	No	3	NO	No	No	No	0	Yes	Yes	No	3	2	A0201	SD*	N/E	Alive
5462	NEGATIVE	315	>100	287	>100	Both	GI	NORMAL	yes	Yes	No	3	NO	No	No	No	0	No	No	No	0	3	A0201	PR	RESPONDER	Alive
5551	UNKNOWN	584	>100	564	>100	Viremia	NA	NORMAL	Yes	Yes	No	4	YES	Yes	Yes	Yes	3	No	No	No	0	5	A0201, B0702	CR	RESPONDER	Alive
5552	UNKNOWN	369	>100	339	>100	Both	CNS, GI	NORMAL	Yes	Yes	Yes	5	YES	Yes	Yes	Yes	3	No	No	No	1	3	A0201	CR	RESPONDER	Alive
5557	UNKNOWN	253	>100	157	>100	Viremia	NA	NORMAL	Yes	Yes	Yes	5	YES	Yes	Yes	No	2	No	No	No	0	3	A0201	PR	RESPONDER	Alive
5560	NEGATIVE	230	>100	191	>100	Both	GI	ABNORMAL	Yes	Yes	Yes	3	YES	Yes	Yes	Yes	3	Yes	Yes	No	3	4	A0201	POD	NON RESPONDER	Died of CMV
5561	NEGATIVE	509	>100	413	>100	Viremia	NA	ABNORMAL	Yes	Yes	Yes	5	YES	Yes	Yes	Yes	3	No	No	No	0	2	A0201 DR0901	CR	RESPONDER	Alive
22230	NEGATIVE	505	>100	22	<100	Organ	CNS	Normal	Yes	Yes	No	4	NO	No	No	No	0	Yes	Yes	No	3	3	A0201 B4402	PR	RESPONDER	Alive
22323	POSITIVE	1953	>100	1945	>100	Organ	CNS	normal	Yes	Yes	No	4	N/A	-	-	-	0	Yes	Yes	No	3	5	B5801 DR 0301	CR	RESPONDER	Alive
2386	NEGATIVE	4940	>100	285	>100	Organ	GI	NORMAL	No	Yes	No	1	N/A	-	-	-	0	No	Yes	No	1	2	A1101, DR0701	CR	RESPONDER	Alive
3619	NEGATIVE	68	≤100	57	<100	Viremia	NA	ABNORMAL	yes	Yes	yes	4	NO	No	No	No	0	yes	No	yes	3	2	N/A	PR*	N/E	Died of other causes
3907	NEGATIVE	406	>100	97	>100	Organ	CNS	NORMAL	Yes	Yes	No	4	NO	No	No	No	0	No	Yes	No	1	2	A0201	PR	RESPONDER	Alive
3951	NEGATIVE	154	>100	141	>100	Viremia	NA	NORMAL	Yes	Yes	Yes	5	NO	No	No	No	0	No	No	Yes	2	2	B4403, DR 1101	CR	RESPONDER	Alive
3972	NEGATIVE	341	>100	323	>100	Both	Lung, CNS	ABNORMAL	Yes	Yes	Yes	4	N/A	-	-	-	0	Yes	Yes	No	3	3	B0702	POD	NON RESPONDER	Died of other causes
3981	NEGATIVE	168	>100	133	>100	Both	GI	ABNORMAL	Yes	Yes	No	4	YES	Yes	No	No	1	Yes	No	No	2	4	A0201	POD	NON RESPONDER	Died of CMV
4062	POSITIVE	407	>100	325	>100	Viremia	NA	ABNORMAL	Yes	Yes	No	3	YES	Yes	Yes	No	2	Yes	Yes	No	2	3	A2601, B3801	PR	RESPONDER	Died of other causes
4124	POSITIVE	72	≤100	20	<100	Viremia	NA	NORMAL	yes	Yes	No	4	YES	Yes	No	No	1	Yes	Yes	No	3	2	B4403,	SD*	N/E	Alive
4232	NEGATIVE	172	>100	155	>100	Viremia	NA	NORMAL	Yes	Yes	No	2	YES	Yes	No	No	1	Yes	No	No	1	3	B3501	POD	NON RESPONDER	Died of CMV
4234	POSITIVE	132	>100	99	<100	Viremia	NA	NORMAL	yes	Yes	No	2	NO	No	No	No	0	Yes	No	No	1	5	B0702	PR*	N/E	Alive
4247	POSITIVE	102	>100	77	<100	Viremia	NA	ABNORMAL	Yes	Yes	No	2	YES	Yes	Yes	No	2	Yes	No	No	3	4	B3502	POD	NON RESPONDER	Died of CMV
4268	POSITIVE	59	≤100	35	<100	Both	GI	NORMAL	Yes	Yes	No	3	NO	No	No	No	0	Yes	Yes	No	5	4	B0702	PR	RESPONDER	Died of other causes
4286	POSITIVE	32	≤100	15	<100	Viremia	NA	NORMAL	Yes	Yes	No	2	NO	No	No	No	0	Yes	Yes	No	3	6	A0201	SD	NON RESPONDER	Died of other causes
4417	POSITIVE	199	>100	182	>100	Both	GI	ABNORMAL	Yes	Yes	No	3	NO	No	No	No	0	No	No	No	0	4	A0201	CR	RESPONDER	Died of other causes
4508	POSITIVE	34	≤100	7	<100	Viremia	NA	NORMAL	Yes	No	No	1	NO	No	No	No	0	Yes	Yes	Yes	4	3	B3508	POD	NON RESPONDER	Died of CMV
4518	POSITIVE	317	>100	283	>100	Viremia	NA	NORMAL	Yes	Yes	No	2	NO	No	No	No	0	No	No	No	0	3	A0201	PR	RESPONDER	Died of other causes
5062	NEGATIVE	148	>100	128	>100	Viremia	NA	NORMAL	No	Yes	No	1	N/A	-	-	-	0	No	No	No	0	2	B3501	POD	NON RESPONDER	Alive
5066	NEGATIVE	278	>100	262	>100	Viremia	NA	ABNORMAL	Yes	Yes	No	3	N/A	-	-	-	0	Yes	No	No	1	2	A0201	PR*	N/E	Died of other causes
5073	POSITIVE	96	≤100	70	<100	Viremia	NA	ABNORMAL	Yes	No	No	1	YES	Yes	Yes	No	2	Yes	Yes	No	3	4	A0201	PR	RESPONDER	Died of CMV
5089	POSITIVE	140	>100	92	<100	Viremia	NA	NORMAL	Yes	Yes	Yes	4	YES	Yes	No	No	1	No	No	No	0	4	A0201	CR	RESPONDER	Alive
5100	NEGATIVE	56	≤100	34	<100	Viremia	NA	NORMAL	No	Yes	No	1	NO	-	-	-	0	No	No	Yes	1	3	B3501	SD	NON RESPONDER	Died of other causes
5208	NEGATIVE	242	>100	214	>100	Both	lung	NORMAL	Yes	Yes	Yes	3	NO	No	No	No	0	Yes	No	No	1	3	B 5301	PR*	N/E	Alive
5265	NEGATIVE	46	≤100	21	<100	Viremia	NA	NORMAL	No	Yes	No	1	N/A	-	-	-	0	No	Yes	No	1	6	A0201	SD	NON RESPONDER	Died of other causes
5335	POSITIVE	98	≤100	92	<100	Viremia	NA	NORMAL	Yes	No	No	2	N/A	-	-	-	0	Yes	Yes	No	2	3	A0201	PR*	N/E	Died Of CMV
5337	POSITIVE	76	≤100	45	<100	Viremia	NA	NORMAL	Yes	Yes	No	2	NO	No	No	No	0	No	No	No	0	4	B4001	POD	NON RESPONDER	Died of CMV
5526	NEGATIVE	195	>100	15	<100	Organ	CNS	NORMAL	yes	Yes	Yes	4	YES	Yes	No	No	1	Yes	No	Yes	3	4	A0101, B 0801	CR	RESPONDER	Alive
5550	NEGATIVE	322	>100	305	>100	Viremia	NA	NORMAL	Yes	Yes	Yes	6	YES	Yes	No	Yes	2	Yes	No	Yes	3	2	A3001, B4101	PR	RESPONDER	Died of other causes
5562	POSITIVE	70	≤100	52	<100	Viremia	NA	NORMAL	No	Yes	Yes	3	N/A	-	-	-	0	No	Yes	No	2	2	DR1101	CR	RESPONDER	Alive
5653	NEGATIVE	110	>100	86	<100	Viremia	NA	NORMAL	No	Yes	No	2	NO	No	No	No	0	No	Yes	No	1	4	A0201	CR	RESPONDER	Alive
5821	NEGATIVE	558	>100	527	>100	Both	GI	NORMAL	Yes	Yes	No	3	NO	No	-	-	0	Yes	Yes	No	2	2	A0101	SD	NON RESPONDER	Alive
5841	NEGATIVE	82	≤100	66	<100	Viremia	NA	NORMAL	No	Yes	Yes	4	No	No	No	No	0	No	Yes	Yes	2	3	A0201	PR	RESPONDER	Died of other causes
5842	NEGATIVE	82	≤100	35	<100	Viremia	NA	NORMAL	Yes	Yes	No	2	N/A	-	-	-	0	No	Yes	No	1	3	B5201	CR	RESPONDER	Died of other causes
5873	NEGATIVE	43	≤100	29	<100	Viremia	NA	NORMAL	Yes	Yes	No	3	NO	No	No	No	0	Yes	Yes	No	2	2	B5301, C0401	PR	RESPONDER	Died of other causes
6048	UNKNOWN	269	>100	247	>100	Viremia	NA	NORMAL	Yes	Yes	Yes	4	YES	Yes	Yes	No	2	Yes	Yes	Yes	3	6	A0201, B0702	POD	NON RESPONDER	Alive
6049	POSITIVE	228	>100	219	>100	Both	GI	NORMAL	Yes	Yes	Yes	5	YES	Yes	Yes	No	2	No	Yes	Yes	3	4	B4403,	POD	NON RESPONDER	Died of other causes
6098	POSITIVE	56	≤100	24	<100	Viremia	NA	NORMAL	Yes	No	No	1	NO	No	No	No	0	Yes	No	No	1	3	A0201	POD	NON RESPONDER	Died of other causes
6106	NEGATIVE	36	≤100	18	<100	Viremia	NA	NORMAL	Yes	No	No	2	YES	Yes	Yes	No	2	Yes	No	No	2	3	A0201	CR	RESPONDER	Alive
6115	POSITIVE	67	≤100	34	<100	Viremia	NA	NORMAL	No	Yes	Yes	3	NO	No	No	No	0	No	Yes	Yes	2	4	B4001	CR	RESPONDER	Died of other causes
21890	POSITIVE	132	>100	119	>100	Both	GI	ABNORMAL	Yes	Yes	Yes	4	YES	Yes	Yes	Yes	3	No	No	Yes	1	4	B0702	SD	NON RESPONDER	Died of CMV
22022	NEGATIVE	173	>100	88	<100	Organ	NA	ABNORMAL	Yes	Yes	No	4	NO	No	No	No	0	Yes	Yes	No	3	7	A0201	PR	RESPONDER	Alive
22072	POSITIVE	112	>100	97	<100	Viremia	NA	NORMAL	Yes	Yes	No	2	YES	Yes	No	No	1	Yes	No	No	1	3	A1101, DR 0701	CR	RESPONDER	Alive
22174	NEGATIVE	39	≤100	35	<100	Viremia	NA	ABNORMAL	Yes	Yes	No	3	NO	No	No	No	0	Yes	Yes	No	3	3	A0201	PR	RESPONDER	Died of other causes
22269	POSITIVE	54	≤100	42	<100	Both	GI	NORMAL	Yes	Yes	No	3	YES	Yes	No	No	1	Yes	Yes	No	4	5	A0201	POD	NON RESPONDER	Died of other causes
22333	NEGATIVE	91	≤100	62	<100	Viremia	NA	normal	Yes	Yes	No	2	NO	No	No	No	0	Yes	Yes	No	3	3	A0201	PR	RESPONDER	Died of other causes
22532	POSITIVE	34	≤100	29	<100	Viremia	NA	ABNORMAL	Yes	Yes	No	4	YES	Yes	No	No	1	Yes	Yes	No	4	3	B3501	POD	NON RESPONDER	Died of other causes
4101	CORD	245	>100	199	>100	Viremia	NA	ABNORMAL	Yes	Yes	No	2	YES	Yes	Yes	No	2	Yes	Yes	No	2	2	A0201	PR	RESPONDER	Died of other causes
4193	CORD	176	>100	150	>100	Both	CNS	NORMAL	Yes	Yes	No	2	NO	No	No	No	0	Yes	No	No	2	2	B5001 DR0701	POD	NON RESPONDER	Died of CMV
5146	CORD	183	>100	127	>100	Viremia	NA	NORMAL	Yes	Yes	No	3	NO	No	No	No	0	Yes	Yes	No	3	1	B3502	POD	NON RESPONDER	Died of other causes
5553	CORD	309	>100	273	>100	Both	CNS, GI	ABNORMAL	Yes	Yes	Yes	3	YES	Yes	Yes	No	2	No	No	No	0	3	A0201	CR	RESPONDER	Died of other causes
5555	CORD	579	>100	218	>100	Viremia	NA	NORMAL	Yes	Yes	No	2	N/A	-	-	-	0	No	No	No	0	5	A0201	SD	NON RESPONDER	Died of Unknown Causes
6072	NEGATIVE	268	>100	228	>100	Viremia	NA	NORMAL	Yes	Yes	No	3	YES	Yes	No	No	1	No	No	No	0	2	A6801	CR	RESPONDER	Alive

Supplemental Table 3. Summary of Risk Factors in Treated Patients

UPN	Source	TCD	HLA MM	Donor SeroNeg	CMV to CMV-CTLs >100 Days	Prior Meds ≥3	Disease	Resistance	Prior aGvHD	IS > CNI, SIRO or MMF	baseline CD4<50 (yes/no)	AAUC >1.5	Number	Response	Best response	Died Of CMV
5554	cBM				Yes	YES		Yes	Yes	No	no		4	Responder	CR	N/A
5558	cBM			Yes		YES			No	No	yes		3	Responder	CR	N/A
5559	cBM		Yes	Yes	Yes	YES		Yes	No	No	no		5	Responder	PR	No
21856	cBM			Yes		YES	Yes	Yes	No	No	n/a		4	Responder	PR	N/A
21968	cBM			Yes		YES			No	No	yes		2	Responder	CR	N/A
22156	cBM		Yes	Yes		YES		Yes	No	Yes	no	YES	6	Responder	PR	N/A
5462	cPBSC			Yes	Yes	YES	Yes		Yes	Yes	no	No	6	Responder	PR	N/A
5551	cPBSC				Yes	YES		Yes	No	No	no		3	Responder	CR	N/A
5552	cPBSC				Yes	YES	Yes	Yes	Yes	No	no		5	Responder	CR	N/A
5557	cPBSC		Yes		Yes	YES		Yes	No	No	no		4	Responder	PR	N/A
5560	cPBSC			Yes	Yes	YES	Yes	Yes	Yes	No	yes		7	Non-Responder	POD	Yes
5561	cPBSC		Yes	Yes	Yes	YES		Yes	No	No	no		5	Responder	CR	N/A
22230	cPBSC		Yes	Yes		YES	Yes		Yes	Yes	yes		7	Responder	PR	N/A
22323	cPBSC					YES	Yes		Yes	Yes	no		4	Responder	CR	N/A
2386	TCD	Yes		Yes	Yes	1	Yes		Yes	Yes	no		5	Responder	CR	N/A
3907	TCD	Yes	Yes	Yes	Yes	YES	Yes		Yes	Yes	no	No	8	Responder	PR	N/A
3951	TCD	Yes	Yes	Yes	Yes	YES			Yes	Yes	yes	No	8	Responder	CR	N/A
3972	TCD	Yes	Yes	Yes	Yes	YES	Yes		Yes	Yes	yes	YES	10	Non-Responder	POD	No
3981	TCD	Yes	Yes	Yes	Yes	YES	Yes	Yes	No	Yes	yes	No	9	Non-Responder	POD	Yes
4062	TCD	Yes			Yes	YES		Yes	No	No	no	No	4	Responder	PR	No
4232	TCD	Yes	Yes	Yes	Yes	2		Yes	No	No	yes		6	Non-Responder	POD	Yes
4247	TCD	Yes				2		Yes	No	No	yes	YES	4	Non-Responder	POD	Yes
4268	TCD	Yes				YES	Yes		No	No	yes	YES	5	Responder	PR	No
4286	TCD	Yes	Yes			2			No	Yes	yes	YES	5	Non-Responder	SD	No
4417	TCD	Yes	Yes		Yes	YES	Yes		No	No	no		5	Responder	CR	No
4508	TCD	Yes	Yes			1			No	No	yes	YES	4	Non-Responder	POD	Yes
4518	TCD	Yes			Yes	2			Yes	No	no	No	3	Responder	PR	No
5062	TCD	Yes		Yes	Yes	1			No	No	no	YES	4	Non-Responder	POD	N/A
5073	TCD	Yes	Yes			1		Yes	Yes	No	yes	YES	6	Responder	PR	Yes
5089	TCD	Yes				YES		Yes	No	No	yes		4	Responder	CR	N/A
5100	TCD	Yes		Yes		1			No	No	yes		3	Non-Responder	SD	No
5146	TCD	Yes	Yes	Yes	Yes	YES			Yes	Yes	no		6	Non-Responder	POD	No
5265	TCD	Yes	Yes	Yes		1			No	No	yes	No	4	Non-Responder	SD	No
5337	TCD	Yes				2			No	No	yes	YES	3	Non-Responder	POD	Yes
5526	TCD	Yes		Yes		YES	Yes	Yes	No	No	yes	YES	7	Responder	CR	N/A
5550	TCD	Yes	Yes	Yes	Yes	YES		Yes	No	Yes	no		7	Responder	PR	No
5562	TCD	Yes	Yes			YES			No	No	no		3	Responder	CR	N/A
5653	TCD	Yes		Yes		2			No	yes	yes	YES	4	Responder	CR	N/A
5821	TCD	Yes	Yes	Yes	Yes	YES	Yes		Yes	YEs	yes		9	Non-Responder	SD	N/A
5841	TCD	Yes	Yes	Yes		YES			Yes	Yes	yes		7	Responder	PR	No
5842	TCD	Yes	Yes	Yes		2			No	No	yes		4	Responder	CR	No
5873	TCD	Yes		Yes		YES			Yes	No	yes	No	5	Responder	PR	No
6048	TCD	Yes			Yes	YES		Yes	yes	Yes	yes		7	Non-Responder	POD	N/A
6049	TCD	Yes			Yes	YES	Yes	Yes	No	No	yes		6	Non-Responder	POD	No
6098	TCD	Yes	Yes			1			No	No	yes	YES	4	Non-Responder	POD	No
6106	TCD	Yes	Yes	Yes		2		Yes	No	No	yes	YES	6	Responder	CR	N/A
6115	TCD	Yes	Yes			YES			Yes	Yes	yes		6	Responder	CR	No
21890	TCD	Yes			Yes	YES	Yes	Yes	No	No	yes		6	Non-Responder	SD	Yes
22022	TCD	Yes	Yes	Yes		YES	Yes		Yes	Yes	yes	YES	9	Responder	PR	N/A
22072	TCD	Yes				2		Yes	No	No	yes	YES	4	Responder	CR	N/A
22174	TCD	Yes	Yes	Yes		YES			No	No	yes		5	Responder	PR	No
22269	TCD	Yes				YES	Yes	Yes	No	No	yes	YES	6	Non-Responder	POD	No
22333	TCD	Yes		Yes		2			No	No	yes	YES	4	Responder	PR	No
22532	TCD	Yes	Yes			YES		Yes	No	Yes	no		5	Non-Responder	POD	No
4101	UCB		Yes			2		Yes	Yes	Yes	no		4	Responder	PR	No
4193	UCB		Yes	Yes	Yes	2	Yes		Yes	No	yes		6	Non-Responder	POD	Yes
5553	UCB		Yes	Yes	Yes	YES	Yes	Yes	No	No	n/a		6	Responder	CR	No
5555	UCB		Yes	Yes	Yes	YES			Yes	Yes	yes		7	Non-Responder	SD	No
6072	UCB		Yes	Yes	Yes	YES		Yes	Yes	Yes	no		7	Responder	CR	N/A
